# Dynamics of Whole Transcriptome Analysis (WTA) and Surface markers expression (AbSeq) in Immune Cells of COVID-19 Patients and Recovered captured through Single Cell Genomics

**DOI:** 10.3389/fmed.2024.1297001

**Published:** 2024-01-31

**Authors:** Jyoti Soni, Partha Chattopadhyay, Priyanka Mehta, Ramakant Mohite, Kishore Tardalkar, Meghnad Joshi, Rajesh Pandey

**Affiliations:** ^1^Division of Immunology and Infectious Disease Biology, INtegrative GENomics of HOst-PathogEn (INGEN-HOPE) Laboratory, CSIR-Institute of Genomics and Integrative Biology (CSIR-IGIB), Delhi, India; ^2^Academy of Scientific and Innovative Research (AcSIR), Ghaziabad, India; ^3^Department of Stem Cells & Regenerative Medicine, D. Y. Patil Education Society, Kolhapur, India

**Keywords:** single cell WTA, AbSeq, COVID-19, recovered patients, immune cell types, FACS

## Abstract

**Introduction:**

Single-cell multi-omics studies, such as multidimensional transcriptomics (whole transcriptomic analysis, WTA), and surface marker analysis (antibody sequencing, AbSeq), have turned out to be valuable techniques that offer inaccessible possibilities for single-cell profiling of mRNA, lncRNA, and proteins.

**Methods:**

We used this technique to understand the dynamics of mRNA and protein-level differences in healthy, COVID-19-infected and recovered individuals using peripheral blood mononuclear cells (PBMCs). Our results demonstrate that compared to mRNA expression, protein abundance is a better indicator of the disease state.

**Results:**

We demonstrate that compared to mRNA expression, protein abundance is a better indicator of the disease state. We observed high levels of cell identity and regulatory markers, *CD3E*, *CD4*, *CD8A*, *CD5*, *CD7*, *GITR*, and *KLRB1* in healthy individuals, whereas markers related to cell activation, *CD38*, *CD28*, *CD69*, *CD62L*, *CD14*, and *CD16* elevated in the SARS-CoV-2 infected patients at both WTA and AbSeq levels. Curiously, in recovered individuals, there was a high expression of cytokine and chemokine receptors (*CCR5, CCR7, CCR4, CXCR3*, and *PTGRD2*). We also observed variations in the expression of markers within cell populations under different states.

**Discussion:**

Furthermore, our study emphasizes the significance of employing an oligo-based method (AbSeq) that can help in diagnosis, prognosis, and protection from disease/s by identifying cell surface markers that are unique to different cell types or states. It also allows simultaneous study of a vast array of markers, surpassing the constraints of techniques like FACS to query the vast repertoire of proteins.

## Introduction

Infectious diseases such as Flu, tuberculosis (TB), Dengue, and respiratory infections like SARS-CoV-2 pose a serious threat to world health. They account for between 25 and 33% of all deaths, making a significant contribution to the global mortality rate (1). In addition to mortality, they stretch healthcare support globally and locally as well. The SARS-CoV-2 outbreak, which led to the COVID-19 pandemic, stands as one of the most significant global pandemics in recent times, with its enduring repercussions potentially yet to unfold (2). Exploring the complex landscape of gene expression is essential for improving our knowledge of these diseases at the molecular level and facilitating the creation of useful diagnostics and targeted therapeutics. Gaining important insights and addressing the issues posed by these infectious diseases requires extensive research in this area. Gene expression encompasses two fundamental stages: transcription and translation, where the genetic information encoded in DNA is converted into functional proteins. RNA and protein levels are believed to be strongly correlated although empirical studies have shown that this link is not always unidirectional (3, 4). According to Schwanhäusser et al., mRNA levels explain only 40% of the variability in protein levels, leaving 60% of the variability unaccountable (5). The observed discrepancies in RNA and protein levels can be traced back to a complex interplay that has numerous complex and dynamic components (6). Post-transcriptional regulation including capping, RNA editing, and alternative splicing plays a requisite role in shaping protein expression and contributes to the differences between mRNA and protein levels (7). These regulatory processes impact RNA stability and quantity, ultimately influencing protein synthesis. The accurate measurement of both RNA and protein expression within different tissues and cells is essential for a comprehensive understanding of disease mechanisms. While significant attention has been given to RNA measurements thus far, it is noteworthy that protein levels frequently exhibit limited correlation with transcript levels (8). Apart from post-transcriptional-and post-translational processes, several other factors can contribute to discordance between mRNA and protein levels. These include long non-coding RNAs, protein and mRNA half-lives and receptor internalization, which can indirectly affect the mRNA and protein levels (9, 10). According to Benoit P. Nicolet, the correlation between mRNA and protein levels is dependent on the specific class of genes and is influenced by factors such as sequence conservation, structure sequences found in the untranslated region of these genes (11). Highly conserved genes tend to show stronger similarities between mRNA and protein expression. On the other hand, less conserved genes may exhibit relatively more discordance between mRNA and protein levels, suggesting additional regulatory mechanisms beyond transcription and translation may impact protein abundance (11). The mRNAs that exhibit differential expression are more likely to exhibit concordance in protein expression than mRNAs that do not exhibit differential expression. In other words, if there is an increase or decrease in mRNA expression, it is more probable that a similar change will be observed in protein expression (12). The precise regulation of these processes is critical for controlling the levels of mRNA and protein in cells and ensuring proper cellular function. Untangling the dynamics between RNA and protein expression in response to infections such as COVID-19, dengue, and tuberculosis is crucial for unraveling the complex mechanisms governing gene expression and protein synthesis. Bridging this gap can lead to the identification of new biomarkers, therapeutic targets, and efficient methods to understand these infectious diseases (8).

Single-cell sequencing studies offer a comprehensive overview of the genes that are actively expressed within a cell. The whole Transcriptome Analysis (WTA) technique, which captures cellular mRNA by its poly-A tail, is the empirical way to study the gene expression at the single cell level, while oligonucleotide-labeled antibody-based techniques, such as AbSeq (Antibody sequencing) and CITE-seq (cellular indexing of transcriptomes and epitopes) enables the simultaneous profiling of transcriptome and cell surface proteins using different barcoding and sequencing approaches (13, 14). Integration of the WTA and AbSeq techniques offers a more comprehensive view of the cellular states which, otherwise could not be investigated using any one of them only. Despite the fact that both mRNA and protein expression are produced by the same cell, most studies consider the differences between mRNA and protein expression, yet the deeper investigation of their disparities remains relatively unexplored (3, 5).

Previously, for assessing the gene/protein expression, researchers used methods including western blotting, transcriptomics, and fluorescence-activated cell sorting (FACS). However, these techniques were limited in their technical abilities and capacity for investigating individual cells. As a result, the development of single-cell technology revolutionized the research field by making it possible to do both surface marker and transcriptome study at single-cell level. This integration of WTA and AbSeq allows for a more comprehensive and detailed understanding of gene expression profiles and protein abundance in individual cell types, facilitating a deeper exploration of cellular heterogeneity and its functional characterization. Single-cell technologies have overcome the limitations of population-level analysis, revealing previously unseen variations and highlighting the significance of mRNA-protein disparities within the cellular systems (15).

In our study, which included healthy, COVID-19 patients as well as recovered, we employed transcriptomics and targeted proteomics to explore the immune cell dynamics, underlying gene expression patterns, and mechanisms of this infectious disease. This enabled us to analyze and compare the gene expression profiles and protein levels in healthy, infected and recovered groups. The findings emphasize immunological aspects of COVID-19 by mapping genes related to immune cell activation, immune system components, and protective immunity. This enhances our understanding of disease-related genomic patterns and helps identify potential modulator genes or proteins. We reported on the differences in cumulative average WTA and AbSeq when studied at the single level and their contribution to cell-to-cell variabilities. We highlighted the functional significance of discordance and concordance between WTA and AbSeq. We observed more discordance within the immune markers at the AbSeq level compared to WTA, which was highly expressed and cell type-specific dynamics. We further clarified the functional relevance associated with these observed discrepancies, which highlighted their importance. Finally, we highlighted the distinct regulation of immune genes and their expression patterns in both normal and infected states, particularly emphasizing the heightened and consistent expression of cell activation markers during infection.

## Materials and methods

### Sample collection and PBMCs isolation

A total of 33 individuals, including healthy, confirmed SARS-CoV-2-infected and recovered, were provided whole blood samples at Dr. D. Y. Patil Medical College, Hospital and Research Institute, Kolhapur, Maharashtra, India (Supplementary Table S1; Supplementary Figures S1A,B). The study cohort was recruited between November and December 2020. All the recovered samples were collected within 1 month after recovery, confirmed by negative real-time reverse transcription–polymerase chain reaction (RT-PCR, Ct value >35) using TRUPCR^®^ SARS-CoV-2 RT qPCR Kit (3B BlackBio Biotech, India, Catalog no: 3B307). Blood samples were collected using BD Vacutainer CPT cell preparation tubes (Becton, Dickinson and Company, United States, Catalog no: 362753), that contained sodium heparin. This method takes advantage of the differential migration of blood cells through polyester gel and a density gradient liquid media during centrifugation. The centrifugation step was performed at room temperature (RT) with a speed of 1800 RCF for 20 min. Following centrifugation, the isolated PBMCs were washed twice with PBS (phosphate-buffered saline, Gibco, United States, Catalog no: 70013032) at 300 RCF for 15 min at room temperature and preserved for future use by cryopreservation in a media composed of 90% Fetal bovine serum-FBS (Gibco, United States, Catalog no: 10082147) and 10% dimethyl sulfoxide (Sigma Aldrich, USA, Catalog no: D8414) (16). This preservation method maintains the long-term viability and integrity of the PBMCs for subsequent analyses.

### Sample processing, single-cell library preparation and sequencing

After thawing and reviving the PBMCs, 2 × 10^5^ cells per sample were used for downstream processing. The cells were labeled with the BD single cell multiplexing kit-human (Becton, Dickinson and Company, United States, Catalog no: 633781) and 40 BD AbSeq antibody oligonucleotides (cell surface marker tagging). Following the manufacturer’s instructions during the labeling procedure ensured precise and effective labeling of the cells with antibodies. The supplementary file contains a list of specific antibodies targeting surface markers, along with their corresponding attached oligos (Supplementary Table S2). Around 30,000 cells were loaded into the cartridge for each experiment. Cell capture beads were loaded onto the cartridge and was followed by the cell lysis step. The poly-adenylated RNA, along with sample multiplexing antibody and AbSeq oligos bind on the oligo-coated beads. Post lysis, the beads were retrieved from the cartridge, and washed before processing to the on-bead cDNA generation. The cDNA was prepared according to the manufacturer’s instructions, and the entire library was generated using the BD Rhapsody WTA amplification kit (Catalog no: 633801, Doc ID: 23–21,752-00). Post cDNA generation, the WTA and the AbSeq and SM (sample multiplexing) products were separated for amplification using specific primers. Post amplification, forward and reverse index were added to the amplified WTA and AbSeq + SM product. Quality and quantity check for libraries were done using Qubit 4 fluorometer (Invitrogen, United States, Catalog no: Q33238) with Qubit High sensitivity double stranded DNA assay (Invitrogen, United States, Catalog no: Q32854), and the Agilent Bioanalyzer 2,100 (Agilent Technologies, United States, Catalog no: G2939BA) with Agilent high sensitivity DNA kit (Agilent Technologies, United States, Catalog no: 5067–4,626). The library was sequenced on a NovaSeq 6,000 sequencing system (Illumina, United States, Catalog no: 20012850) using S2 sequencing reagent kit (Illumina, United States, Catalog no: 20028315) with 101 paired end cycles, achieving a sequencing depth of approximately 30,000 reads per cell for WTA and 500 reads per AbSeq per cell.

### Single-cell RNA seq data processing and clustering

**Figure 1:**
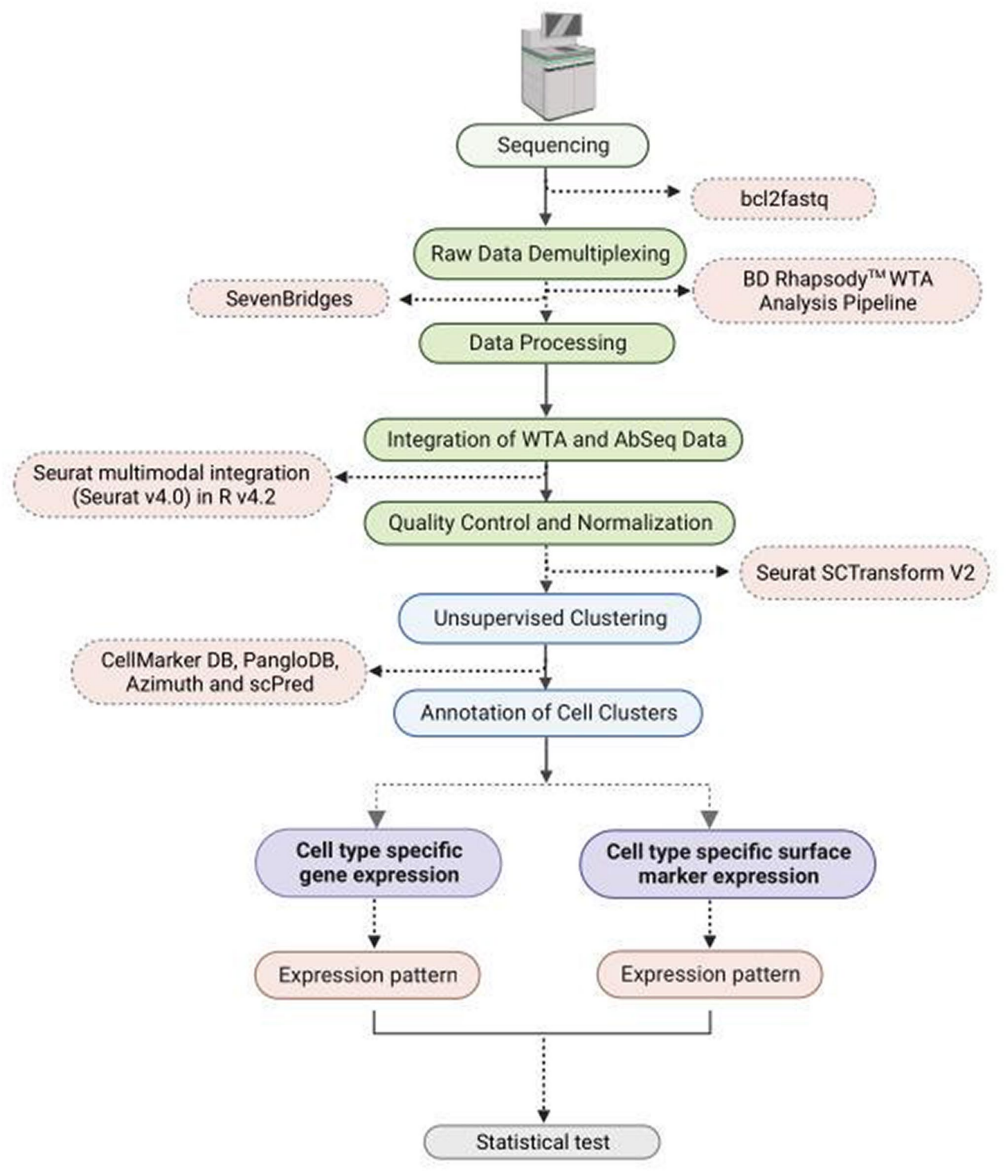
Flowchart depicting methodology steps for understanding WTA/AbSeq concordance and discordance using single cell RNA Seq technology.

The raw sequencing data was demultiplexed using bcl2fastq.^1^ The sequencing data (FASTQ format) was uploaded to SevenBridges platform for further analysis using BD Rhapsody™ WTA Analysis Pipeline (Figure 1). The pipeline takes the FASTQ files, a reference genome file, and a transcriptome annotation file for gene alignment. The pipeline generates molecular counts per cell, read counts per cell, metrics, and an alignment file. The count matrix was then processed using Seurat v4.0 in R v4.2 for quality check (QC), normalization, clustering, and cell type annotation. For AbSeq analysis an AbSeq reference (FASTA format) was generated using BD AbSeq reference generator.^2^ This was used along with the FASTQ file, the genome reference and transcript annotation in BD Rhapsody™ WTA Analysis Pipeline to generate a combined count matrix for WTA and AbSeq. The expression of the AbSeq and the corresponding genes were fetched from the annotated Seurat Object for targeted statistical analysis and visualization. The AbSeq count matrix was separated from WTA count matrix and was integrated to the SeuratObject of WTA data following the Seurat multimodal integration guide.^3^

At the outset of the study, 163,197 cells were included, and the count matrices derived from both WTA and AbSeq experiments were integrated. To enhance the overall quality of the dataset, reduce technical noise, maintain data integrity, and ensure that downstream analyses, cells with a Unique Molecular Index (UMI) count exceeding >2,500 and below <20 were excluded, while batch effects were rectified through normalization procedures (17, 18). The data were further normalized using Seurat scTransform V2. Unsupervised clustering was performed with a resolution of 0.4 to group cells, and the results were visualized using the t-SNE algorithm. The cluster-specific differential expression DE genes were identified using the FindAllMarker function of the Seurat package. Clusters were comprehensively annotated by employing a range of tools, such as CellMarker DB (19), PangloDB (20), Azimuth (21), and scPred, using both manual and automatic methods. Genes and markers list used to identify specific cell types are mentioned in Supplementary Table S3. The previous publication provides a comprehensive methodology description (18). All the codes used in the study are available at Zenodo.^4^

### Statistical analysis

Wilcoxon signed rank test was performed in R studio (version 4.2.1) to calculate significance (Sample wise count matrix mentioned in Supplementary Tables S4A,B) within WTA and AbSeq normalized counts across healthy, infected, and recovered individuals which is highlighted in Supplementary Tables S5A,C for WTA and Supplementary Tables S6A,C for AbSeq.

## Results

### Differential expression of immune cell markers at WTA and AbSeq levels

**Figure 2:**
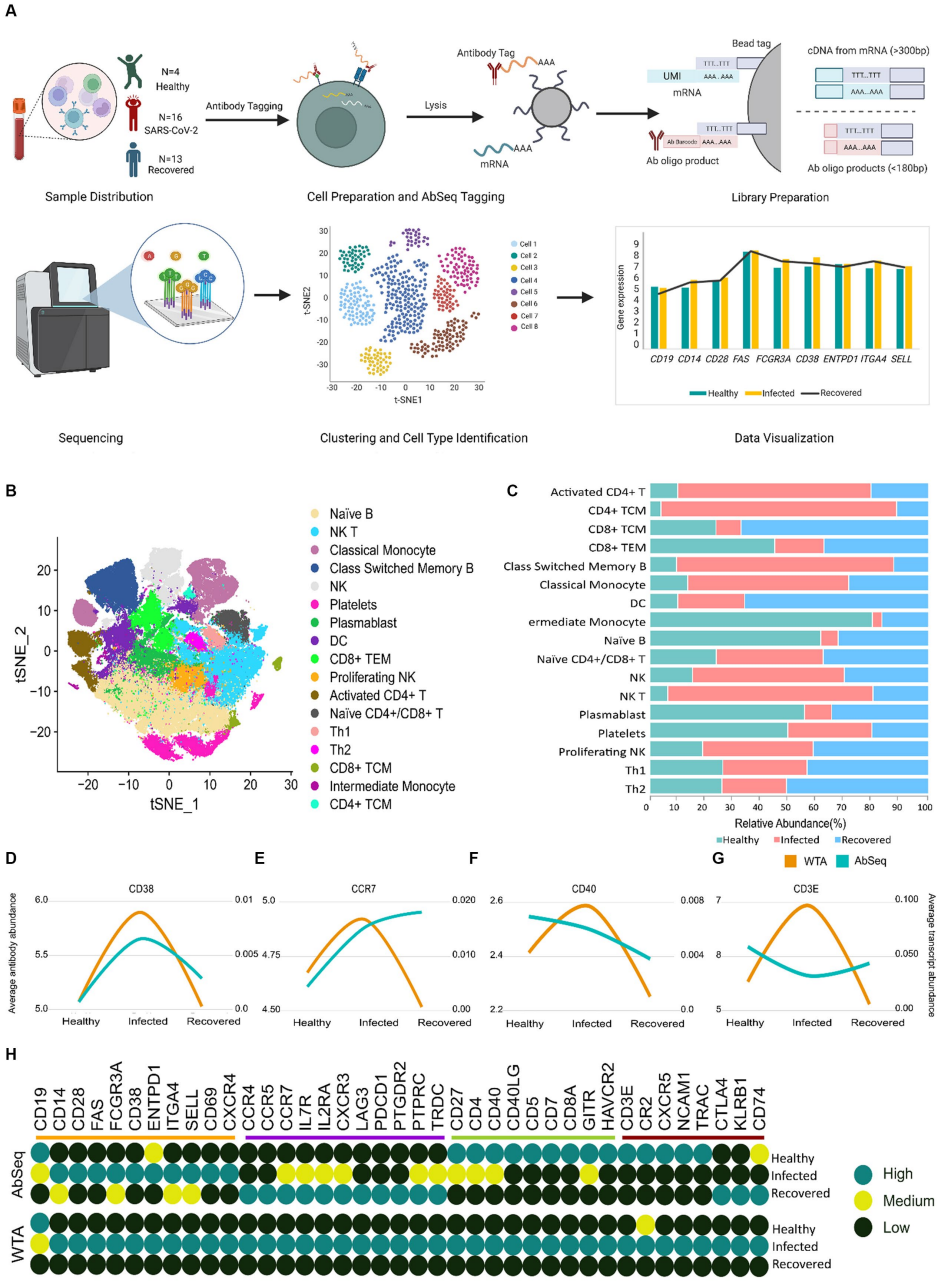
Correlation of WTA/AbSeq across healthy, SARS-CoV-2-infected and recovered individuals. **(A)** Sample distribution across the three groups of Healthy, SARS CoV-2 Infected and Recovered, schematic workflow followed by library preparation and scRNA sequencing for WTA and AbSeq. **(B)** tSNE visualization of the 124,726 cells across the three groups. **(C)** The stacked bar plot shows the relative abundance of the cell types across the three groups. **(D–G)** Four different patterns were observed across the 39 genes. **(H)** Distribution of all 39 immune genes with their expression patterns and comparison groups WTA/AbSeq.

Previous studies have highlighted the discrepancy between mRNA and protein levels across various tissues and cells (22, 23). In our single cell-based investigation, we observed differences between protein markers and their corresponding mRNA levels. Using PBMCs from SARS-CoV-2-infected (*n* = 16), Recovered (*n* = 13), and Healthy (*n* = 4) individuals, we performed single-cell multi-omics study (WTA and AbSeq) using the BD Rhapsody express single cell analysis to understand the immune response dynamics during and post SARS-CoV-2 infection (Figure 2A). The clinical and demographic details of the individuals are available at Supplementary Table S1. The age and gender matched patients recruited in our study were chosen to minimize inter-individual differences (Supplementary Figures S1A,B). We further focused on two key subsets of information about their disease state: HRCT score and C-reactive protein (CRP) levels. Both CRP and HRCT scores were used to categorize the patients into different disease states. To ensure a more homogeneous group, we employed a statistical method called a Student’s *t*-test to analyze and represent the data using a box plot. We have also checked for the immunological response within the Infected group. We used C-reactive protein (CRP) expression as a marker for immunological response and found no significant difference between the different severity and age groups among the infected individuals. Initially, 163,197 cells were identified post-sequencing from all 33 samples. Following QC and the removal of low-quality cells, 124,726 cells were retained. Batch correction and normalization were done using Seurat SCTransform v2; followed by dimension reduction and unsupervised clustering (using the Louvain algorithm). The clusters were annotated both manually (canonical markers from CellMarker, PangloDB, and Azimuth) and Support Vector Machine based tools (scPred).

Our study revealed the presence of 17 distinct clusters across the Healthy, Infected, and Recovered, indicating variations in the abundance of different cell types (Figures 2B,C). We subsequently looked for the WTA and AbSeq profiles to gain insight into gene expression patterns and protein abundance across different cell types. Through this analysis, we explored the expression patterns of these markers to understand the molecular diversity and functional characteristics of the individual cells, across the groups and examined the variations within each group. The significance of expression difference (both WTA and AbSeq) across groups are available at Supplementary Tables S5, S6. We performed an expression pattern analysis on the WTA and AbSeq, dividing them into two main categories: *similar* and *dissimilar*. In the *similar* category, we observed parallel expression trajectories between WTA and protein levels. For example, if the WTA expression level was moderate in the healthy group, the corresponding AbSeq expression level was also moderate. Similarly, if the WTA expression level was high in the SARS-CoV-2-infected group, the AbSeq expression level was also high (Supplementary Figure S2). On the other hand, in the *dissimilar* category, we found that the WTA and AbSeq patterns did not follow the same trajectory between the groups. Interestingly, we noticed that the WTA expression pattern remained unchanged for all 39 genes within a given group. However, we observed four different patterns at the surface marker level, indicating variations in protein expression despite the stable WTA expression pattern. A group of surface markers (*CD38, CD28, CD69, CD14, FCGR3A, ENTPD1, SELL, FAS, CXCR4, ITGA4*, and *CD19*) exhibited expression pattern similar to the corresponding mRNA expression (Figure 2D). Another set of surface markers (*CD4, CD8A, CD5, HAVCR2, CD40LG, CD27, CD7, GITR*, and *CD40*) exhibited an expression pattern opposite to their WTA counterparts in the Healthy group but had a similar expression in other groups (Figure 2E). A third group of surface markers (*TRDC, CCR7, PTPRC, LAG3, IL2RA, CCR5, PDCD1, PTGDR2, CXCR3, CCR4*, and *IL7R*) exhibited expression pattern opposite to their WTA counterparts in the Recovered group but had similar expression pattern in the other groups (Figure 2F). Finally, 8 surface markers (*CXCR5, CR2, NCAM1, KLRB1, CD3E, CTLA4, TRAC*, and *CD74*) had expression pattern completely opposite to their WTA counterparts across the three groups (Figure 2G). Overall, about 72% of the surface markers showed discordance, while remaining 28% exhibited concordance with their corresponding mRNA expression (Figure 2H). The specific expression patterns for the surface markers as well as the expression profile of AbSeq and WTA are available as Supplementary Figures S3–S6 and Supplementary Table S7.

### Functional relatedness of WTA and AbSeq resulting in differential expression patterns

**Figure 3:**
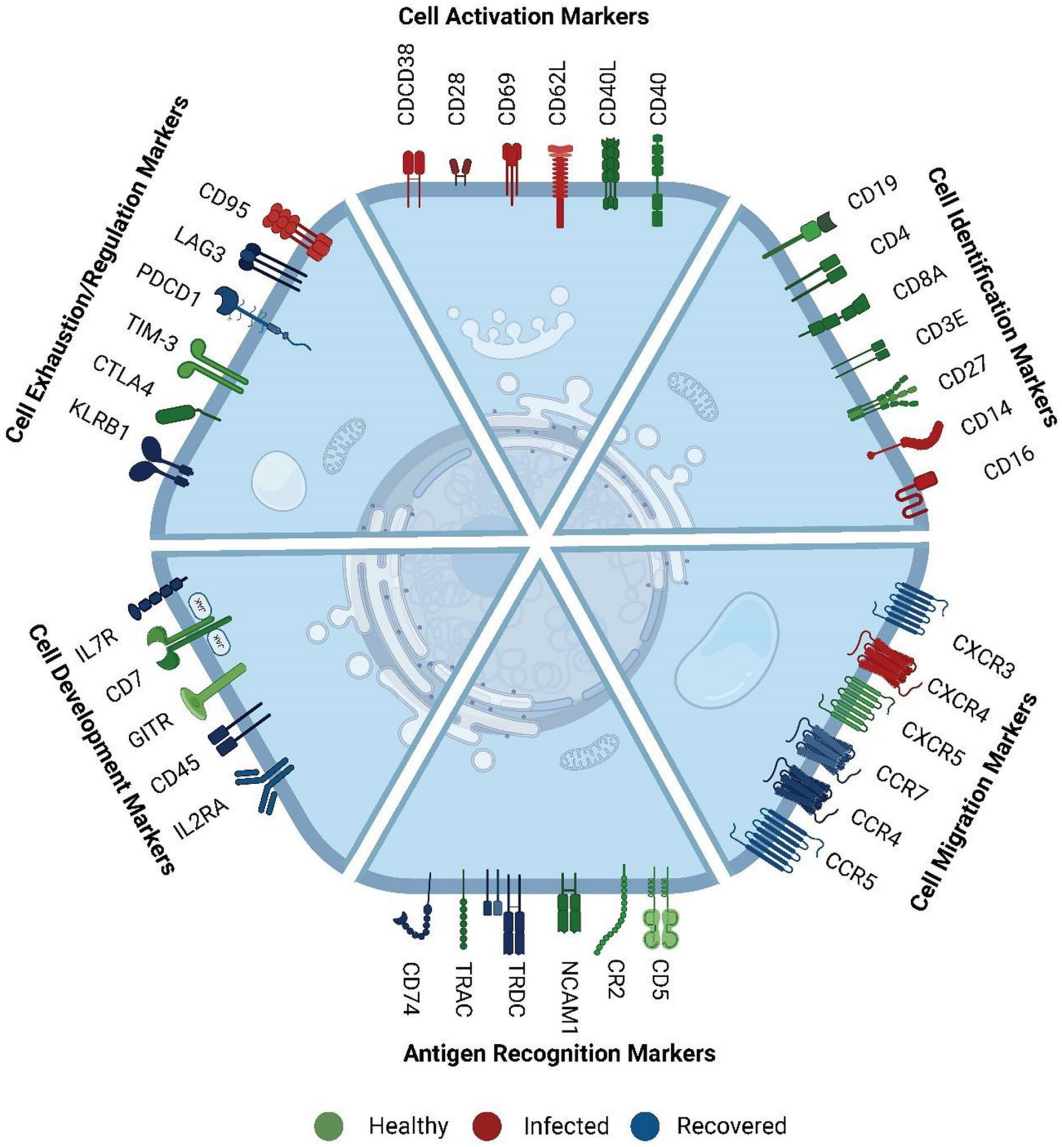
Functional role of surface markers. These markers were mainly involved in cell activation, cell identification, cell migration, antigen recognition, cell development and proliferation, cell exhaustion, and regulation markers. The color represents their comparatively higher abundance across the three groups. Green color markers represent abundant markers within healthy individuals. Red color represents markers high in SARS-CoV-2 infected individuals, whereas blue markers indicate abundance in the recovered. The cell activation markers were high in infected, exhaustion markers were high in recovered, and cell identification and development markers were high in healthy.

Subsequently, we investigated the functional implications of genes across healthy, infected, and recovered individuals, driven by the hypothesis that the presence or absence of correlation between mRNA and protein levels for a gene may be indicative of its functional role (Figure 3). We made an intriguing observation that genes exhibiting similar expression patterns also displayed functional relatedness. Notably, certain cell surface markers (*CD38, CD28, CD14, ENTPD1,* and *SELL*) showed concordance between their mRNA and protein levels. These markers are known as *cell activation markers*, and their elevated expression in SARS-CoV-2-infected individuals signifies their upregulation during infection (24–26). During SARS-CoV-2 infection, an observed increase in the abundance of specific immune cell subsets, including subsets of T cells, B cells, NK cells, and monocytes, that express these markers has been noted (Figure 2C). This suggests a potential involvement of these cell populations in the immune response and highlights their relevance in the context of infection.

We further observed that *cell migration and cell exhaustion-related surface markers* (*CCR4, CCR5, CCR7 (CD197), IL7R (CD127), IL2RA (CD25), CXCR3, LAG3, PDCD1 (PD-1)*) exhibited high expression levels in recovered individuals. Chemokines are essential in the immune response to viral infections as they play an important role in facilitating both innate and adaptive immune cells to the sites of infection. They also enhance these cells’ capacity to synthesize antiviral compounds and exert cytotoxicity, which strengthens the body’s general resistance to viral infections. The level of concordance or discordance can provide insights into the regulation and functional relevance of specific genes in various biological processes.

Conversely, markers related to *cell identity and cell regulation* such as *CD8A, CD3E, CD5, CD7, CXCR5, NCAM1 (CD56), TRAC* and *GITR, HAVCR2 (TIM-3), CR2, CTLA4, KLRB1, CD74* (27) displayed higher expression in healthy individuals compared to the infected and recovered patients. While the overall abundance of cells expressing these markers was decreased in the recovered individuals, it is likely that the remaining cells have upregulated the expression of these markers as a means of promoting robust recovery. Higher expression of these markers in healthy individuals suggests a responsive immune system with regulated immune response. Conversely, decreased expression or altered patterns of these markers can indicate immune dysregulation or dysfunction associated with various diseases or conditions. We have reviewed the specific function of the surface markers, their role during infection and cell type specific expression in Supplementary Table S8. This suggests that the different AbSeq expression patterns observed are associated with specific biological functions rather than any sporadic burst.

### Immune cells with a particular function have different patterns between WTA and AbSeq

Within cell types, we looked at the WTA and AbSeq expression patterns through integrative analyses. We could detect heterogeneity in the expression of markers within the cell populations under various circumstances. It is interesting to note that when we looked at the cumulative average at the WTA level, most cells displayed comparable expression patterns (28). However, contrasting expression has been observed at AbSeq level (Supplementary Figure S7). To elucidate the underlying mechanisms responsible for the discordance observed in the AbSeq data, we analyzed the variability in both cell type-specific marker expression and cell type abundance across the three groups. Based on our observations, it can be inferred that the discordance detected in the AbSeq data can be predominantly attributed to individual cell types including activated CD4^+^ T cells, CD4^+^ TCM, CD8^+^TCM, CD8^+^TEM, Classical Switch Memory B cells (CSMB), Classical Monocytes, Dendritic Cells, Intermediate Monocytes, Naive B, Naive CD4^+^/CD8^+^ T cells, NK cells, NK T cells, Plasmablasts, Platelets, T helper1 and T helper 2 cells.

#### Consistency of immune cell identities in healthy individuals

**Figure 4:**
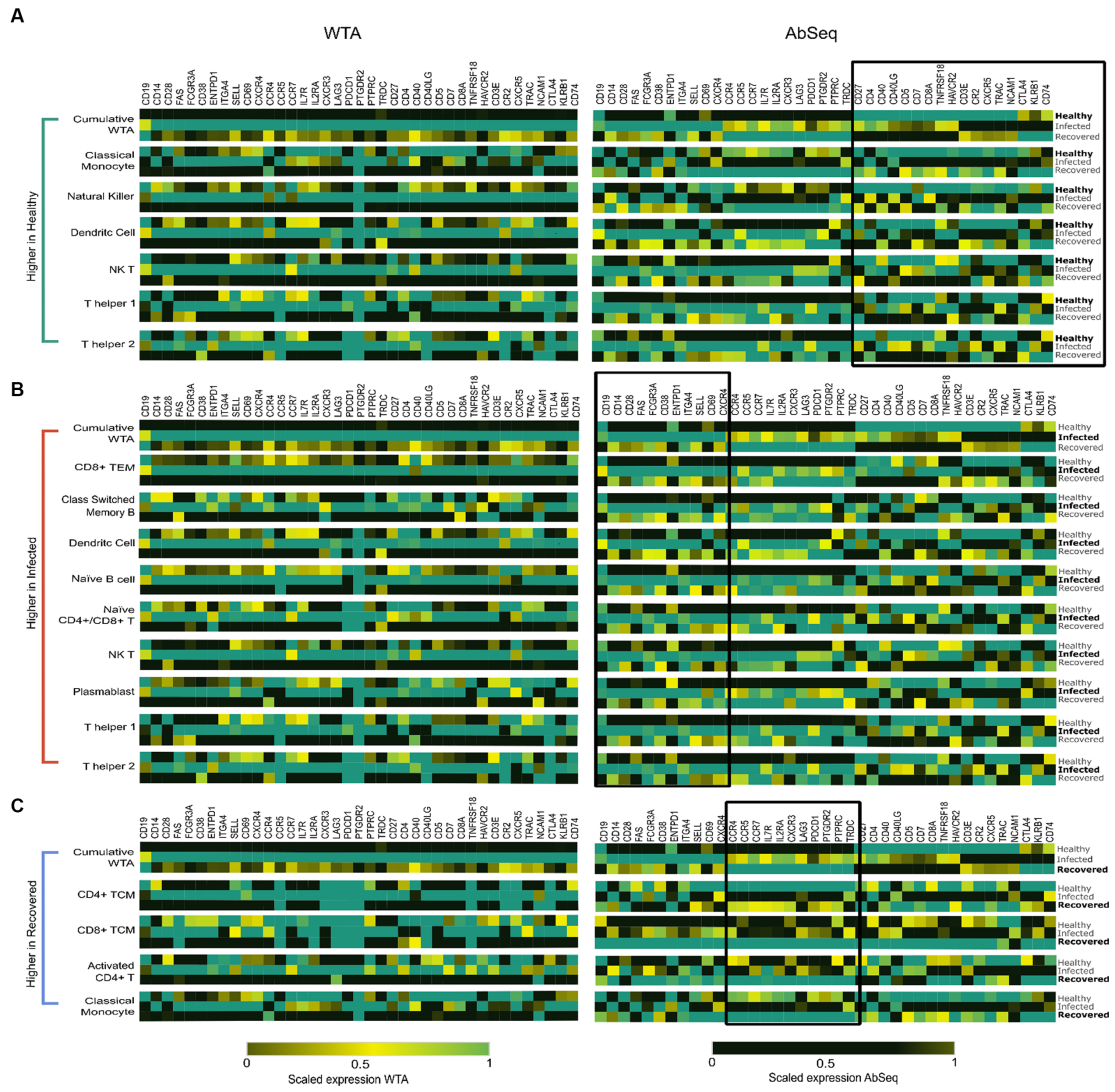
Highlights the specific cell types contributing to the distinct expression patterns observed at the AbSeq level. **(A)** High expression of specific markers in Dendritic cells (DCs), Classical Monocytes (CMs), Natural Killer (NK) cells, Natural Killer T cells (NKT), T helper 1 cells, and T helper 2 cells are primarily responsible for the elevated expression of cell identity markers and co-receptor surface markers in healthy individuals, as highlighted on the right side of figure. **(B)** Cell types including CD4^+^ T Effector Memory cells, Classical Switch Memory B (CSMB) cells, Dendritic cells (DCs), Naive B cells, Naive CD4^+^/CD8^+^ T cells, Plasmablasts, Natural Killer cells, and T helper 1 and T helper 2 cells. These cell populations collectively contribute to the elevated expression of cell activation markers and exhibit a concordance between mRNA and protein levels. **(C)** Activated CD4^+^ cells, CD4^+^ T Central Memory cells, CD8^+^ T Central Memory cells, and Classical Monocytes exhibit heightened cytokine and chemokine receptor expression in recovered individuals.

Our study revealed a decrease in the expression of cell identity markers and co-receptors in individuals who were either infected for SARS-CoV-2 or recovered when compared to the healthy individuals. This decrease was observed primarily in specific cell types, such as dendritic, natural killer, natural killer T, classical monocytes, and T helper 1 and T helper 2 cells (Figure 4A). Our results suggest that healthy individuals maintain a state of balanced innate and adaptive immunity, which is clear in the high expression of cell identity markers and co-receptors. These markers may play a critical role in immune cell activation and subsequent immune response.

#### The concordance of cell activation markers in SARS-CoV-2-infected individuals

Our data revealed a significant high expression of *CD14, FCGR3A, CD38, CD28, CD69*, and *CD62L* in the SARS-CoV-2-infected patients. Studies have reported that these markers are commonly associated with cell activation (29–31). Upon further investigation of the cell types expressing these markers, we observed that Classical Switched Memory B (CSMB) cells, Dendritic cells (DC), Naive B cells, Plasmablasts, Natural killer T (NK T) cells, CD4^+^ T cells, Naive CD4^+^/CD8^+^ T cells, as well as T helper 1 and T helper 2 cells were predominantly involved (Figure 4B). They are essential for eliciting immunological responses such as antibody formation, cytotoxicity, and cytokine release, to configure infections and safeguard the host.

#### Elevated cytokine and chemokine receptor expression in recovered individuals: consequences for cytokine storm response

The expression of markers associated with cell migration and exhaustion, namely *CXCR3, CCR7, CCR5, CCR4, LAG-3,* and *PDCD1*, was observed to be elevated in individuals who had recovered from SARS-CoV-2 infection (32). Notably, these markers were predominantly expressed in activated CD4^+^ and CD4^+^ TCM cells, CD8^+^ TCM cells, and classical monocytes (Figure 4C). These cell types contribute to the immune memory and defense mechanisms in recovered individuals, helping to prevent possible reinfection and fostering a quicker and more efficient response to pathogens (Supplementary Table S9). The observed variations in marker expression collectively suggest the presence of a robust and efficient immune response marked by the persistence of long-lasting memory T cells, indicating recovery of patients. This finding highlights the critical role of an effective immune response in providing immunity against infectious agents.

## Discussion

Despite extensive research on the relationship between mRNA and protein expression, the underlying mechanisms that lead to their concordance or discordance remain incompletely understood. Understanding the intricate relationship between mRNA and protein levels is of utmost importance for addressing crucial biological states, especially during infections. This encompasses unraveling the complexities of immune responses in steady-state conditions, diseased states, and during the process of recovery, while also considering the inherent heterogeneity within immune cell populations. Such investigations hold great promise in advancing our comprehension of immune system dynamics and its implications in health and disease toward future pandemic preparedness.

In response to infections, protein level undergoes dynamic variations that are primarily fueled by changes in mRNA abundance, especially for genes involved in immune responses (4). Our study employed the WTA and AbSeq techniques using BD Rhapsody to investigate a range of physiological conditions: healthy, infected and recovered. The mRNA and protein levels are often averaged across several cells when analyzing in bulk level and in steady-state conditions, ignoring any differences that may exist between individual cells. Our dataset brings attention to a collection of genes that demonstrate clear discordance between mRNA and protein expression levels at the individual cellular level in PBMCs. While the baseline of surface marker and their corresponding RNA expression are different because they are two measurements at two different levels but at the same time: one within a cell, and the other on the cell surface. Given the different biological regulation during transcription and translation, their baseline of expression is expected to be different. It is also true that RNA within a cell have multiple regulators as compared to a relatively stable surface protein which in turn can lead to more variations in the RNA counts as compared to the surface markers. The fact that the AbSeq expression was not so different at the group level (Supplementary Table S7) but at cell type level (Supplementary Table S4), it reiterates our findings that the discordant expression of WTA and AbSeq are plausibly cell type specific. A study by Zerdes et al. also found discordance in protein and RNA expression levels of PD-L1 in breast tumor samples (33). Furthermore, our results align with previous studies by Liu et al. and Buccitelli et al., which employed high-throughput sequencing methods, including bulk RNA-seq (8, 34). These studies also identified substantial gene groups demonstrating disparities in mRNA and protein expressions in their respective datasets. This accumulation compromises the fundamental distinctions between cells and their functional importance. In other words, tissues in general are made up of various cell types that communicate with one another. Because of this, heterogeneity within a single tissue will have radically different transcriptome and proteome compositions, both qualitatively and statistically (35). This phenomenon may be attributed to the intermittent nature of transcription, where RNA synthesis occurs in sporadic bursts with relatively shorter lifespan of RNA molecules. In contrast, proteins tend to be more stable and play vital roles in carrying out cellular functions (36, 37). Several factors, such as post-transcriptional and translational modifications, RNA and protein half-life, alternative splicing, and cellular states, may influence this regulation.

The findings from our study suggest a possible multi-layered relationship between mRNA and protein levels by showing that the rise in cell activation markers at both the WTA and AbSeq levels reflects their critical function in inducing a quick response to infections (24). Interestingly, *CD38*, which is known to confer protection against bacterial and parasitic pathogens, was found to be upregulated in the study. *CD28*, a cell surface receptor, has been recognized as a significant marker of immune activation due to its vital role in promoting the proliferation and maintenance of T helper cells during viral infections. T helper cells play a crucial role in orchestrating immune responses by activating other immune cells and aiding in the clearance of pathogens. *CD28* engagement provides essential co-stimulatory signals that enhance T cell activation and effector functions, leading to an effective immune response against viral infections. The identification of *CD28* as a potential marker of immune activation highlights its importance in providing crucial signals for the optimal functioning of T helper cells during infections, thereby contributing to the overall immune defense against viral pathogens (38, 39). *CD39*, a cell surface marker expressed on various immune cells such as B cells, monocytes, and *CD4^+^* T cells, has emerged as a significant activation marker with regulatory properties. During infections, *CD39* plays a crucial role in modulating immune responses and maintaining immune homeostasis. It functions as an ectonucleotide, catalyzing the hydrolysis of ATP and ADP into AMP and subsequently into adenosine. By converting ATP and ADP, which are pro-inflammatory molecules, into adenosine, *CD39* helps regulate immune activation and dampen excessive inflammation. Similar to our findings, a study reported that certain cell surface markers, including *CD8a, CD11b, FCGR3A (CD16), CD19, CD20, and CD25,* exhibited a strong correlation between their mRNA and protein expression levels, suggesting their potential as cell identification markers.

Evidence from a parallel study highlights discrepancies between mRNA and protein levels of several markers, such as *CD3, CD4, CD27, CD34, CD69*, and *CD80*. These findings indicate the likelihood of variations in post-transcriptional and translational modifications among different genes (40).

These findings shed light on the complex interplay between immune cells and the pathogens they encounter, providing new insights for developing targeted therapies (41). The downregulation of antigen recognition and presentation markers indicates a discrepancy between mRNA and protein expression levels (42). Viruses have evolved diverse mechanisms to evade or suppress immune responses, one of which involves targeting and depleting cell surface major histocompatibility complex (MHC) molecules. MHC molecules play a critical role in presenting viral antigens to immune cells, enabling the recognition and elimination of virus-infected cells by the immune system. By depleting cell surface MHC molecules, viruses impair the ability of the immune system to detect infected cells and mount an effective immune response (43). This suggests viral suppression of mRNA translation, subverting the host’s immune response. These observations align with previous reports of downregulation of antigen-presenting markers observed in both SARS-CoV-2 and other infectious diseases, indicating a novel mechanism employed by the virus to evade cellular immunity (44, 45).

**Figure 5:**
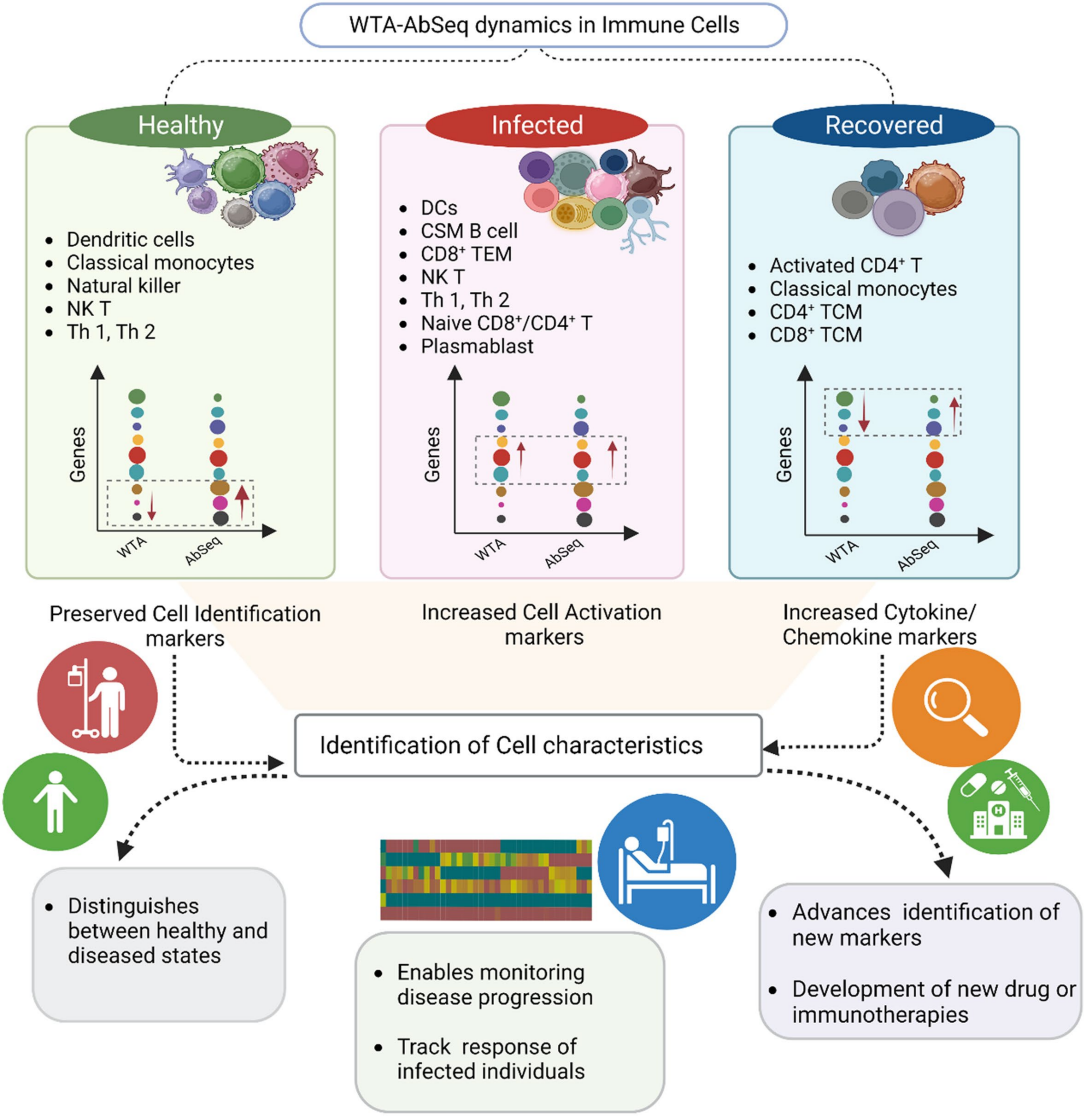
Summary of the key findings from the study. The graphical visualization illustrates the differences between WTA and AbSeq within distinct cell types across healthy, infected, and recovered groups. The gene expression graph represents various genes (circle color) with diverse expressions (circle size) in WTA and AbSeq groups. Notably, our findings highlight the importance of a multi-omics approach in enhancing disease diagnosis and prognosis.

Increased expression of chemokine and cytokine receptors such as CXCR5, CCR4, CCR5, and CCR7 has been reported in both active and recovered COVID-19 individuals (18, 46). In our results we observed high expression of cytokine and chemokine receptors in the recovered individuals majorly in activated CD4^+^ T cells, CD8^+^ T central memory, CD4^+^ T central memory cells (Figure 5). The observed elevation in cytokine and chemokine activity markers among individuals who have recovered could be attributed to a decrease in the expression of *CD40* and *CD40LG*, which are essential regulators of the cytokine/chemokine response. Additionally, another possible factor contributing to the heightened cytokine response is the depletion of a specific subset of Naive CD4^+^ T cells that express genes such as BACH and MALAT1, as discussed in our previous study. Notably, the increased cytokine/chemokine was mainly observed in the Activated CD4^+^ T cell, CD4^+^ TCM, Classical monocyte and NK cell, despite reduced expression of activation markers in the recovered individuals. The activation of T cells, dendritic cell migration to lymph nodes for antigen presentation, and the development of immune responses against pathogens are all influenced by chemokine receptors like *CCR7*. Similarly, *CCR5* primarily expressed on T cells, macrophages, and dendritic cells. Additionally, *CCR5* is involved in immune cell recruitment to inflammatory sites and plays a role in immune surveillance and defense against pathogens. *CCR4* is also associated with Treg migration to tissues and immune tolerance, contributing to the regulation of immune reactions and prevention of excessive inflammation. IL7R signaling is essential for T and B cell proliferation, survival, and differentiation which contribute to the adaptive immune response (47, 48). Intriguingly, these markers exhibit low expression at the WTA level but high expression during AbSeq in recovered group, suggesting that the cells may have successfully normalized the transcriptional activity of these genes during recovery, while their protein levels remain elevated, indicating a sustained need for these receptors. Some marker exists in both membrane bound as well as soluble form such as *IL2RA*. The soluble form of *IL2RA* is generated through alternative splicing events and cleavage of the bound receptor, leading to the release of its extracellular domain. Soluble IL2RA acts as a decoy receptor, competing with the membrane-bound form for ligand binding. This competition attenuates signaling, stabilizes ligands, and can provide additional signaling through interactions with other cell surface proteins (49). Although soluble receptors do play an important role in disease outcomes, they remained uncaptured using techniques which only captures surface markers and hence represents a special challenge for these technologies. It emphasizes the importance of integrating transcriptomic and proteomic data to gain a comprehensive understanding of the immune response by comparing the immune profiles of healthy, infected, and recovered persons, the integration of both WTA and AbSeq analysis approaches can aid in the identification of immunological markers linked to disease development and recovery. The dynamics of the immune response, such as variations in immune cell populations, activation of immunological pathways, and generation of cytokines and other immune-related chemicals, are also important to understand. Hence, WTA and AbSeq data should be examined together, ideally rather than separately.

AbSeq distinguishes itself as a very effective surface marker study method, providing multiplexing capabilities, quantitative results, high sensitivity, and flexibility in the marker selection. AbSeq complements and is even advantageous over the flow cytometry, in particular, for single cell-based research, where surface marker information plays a crucial role in annotating and identifying cell clusters. Compared to the AbSeq approach, FACS possesses limitations in identifying various surface markers pertinent to disease and host response. FACS is restricted by the availability of specific fluorescent-labeled antibodies, limiting the simultaneous analysis of markers, especially rare or newly discovered ones. To characterize surface markers, AbSeq provides a more thorough and high-throughput method. It combines the effectiveness of next-generation sequencing with the strength of antibody-based profiling, allowing the simultaneous identification of several markers at the single-cell level. In the near future, AbSeq and FACS will likely complement one another, allowing researchers to learn more about the intricate workings of the immune system and how it contributes to numerous infectious diseases. While the study is based on 33 samples, the findings are derived from ~1,24,000 cells (~3,800 cells/sample), which is a significant number considering other limited research in the similar emerging domain of infectious disease understanding (40, 50, 51). However, more research must be undertaken to determine, (i) the effectiveness of AbSeq in locating the rare and multifaceted surface markers that may be relevant to the disease, and (ii) whether it is helpful to understand its effectiveness in assessing disease severity and its potential role in providing protection during infection? Empirical studies have highlighted the discrepancy between surface marker expression and mRNA expression time and again, however, there is a lack of information about the reason for the discrepancy between surface marker expression and mRNA expression. In this present study, we intend to deconvolute the surface marker and mRNA expression profile with respect to the cell type vis-a-vis groups (Healthy, Infected, Recovered). While we have considered the potential reasons for the discrepancies based on existing literature, our own findings indicate that the observed differences are specific to cellular-level variations associated with the various health states and their functional relevance. Our findings advocate for AbSeq to be a better alternative to FACS-based understanding of surface markers and suggest integrative understanding of the surface marker and mRNA expression vis-a-vis cell types and disease status for a comprehensive understanding of the cellular events. Undertaking a comprehensive analysis of the complex interaction between transcriptional and translational processes has the immense potential to generate deeper and innovative insights into the regulation of cellular events (such as post-transcriptional regulation, translation efficiency, and cellular responses to stress, alternative splicing, protein turnover, and disease-related pathways). Further investigation should be expanded in order to explore possible associations with mechanisms of regulation. Additionally, a thorough examination of regulatory components, such as modifications occurring after transcription and pathways involving protein degradation, could offer a more thorough comprehension of the observed variations. By adopting an integrative approach, we can potentially enhance our understanding of the dynamics within cells and establish a foundation for focused inquiries into therapeutic interventions or strategies of precision medicine.

## Conclusion

In conclusion, our single-cell study has yielded significant insights into the dynamic nature of gene expression and regulation within individual cells. Cell type-specific protein and RNA dynamics offer valuable insights into the gene regulation, cellular heterogeneity, and disease mechanisms. The generation of proteins, the ultimate functional output, is not only dictated by mRNA levels; hence it is crucial to acknowledge that relying solely on transcriptomics data may lead to biased findings. Therefore, further exploration in this field is essential to unravel the functional significance of the observed discordance between mRNA and protein levels in various physiological states of the body. With single-cell-based AbSeq approach, thousands of markers can be profiled, circumventing the drawbacks of fluorescence-based sorting techniques like FACS. With no restrictions on color, this cutting-edge method provides a more thorough and precise understanding of cellular markers. Although we only included 39 surface markers, there is a need to look over this dynamicity in mRNA and protein using more surface proteins to gain an insight into the exact function of the cells under different conditions. Additionally, such a study would contribute to refining analytical pipelines, considering these dynamics, and ultimately improving cell type annotation for more accurate inferences derived from single cell.

## Data availability statement

The original contributions presented in the study are publicly available. This data can be found in the NCBI GEO repository, accession number GSE201088: https://www.ncbi.nlm.nih.gov/geo/query/acc.cgi?acc=GSE201088.

## Ethics statement

The studies involving human participants were reviewed and approved by CSIR-IGIB’s Human Ethics Committee Clearance (Ref No: CSIR-IGIB/IHEC/2020–21/01). The studies were conducted in accordance with the local legislation and institutional requirements. The participants provided their written informed consent to participate in this study.

## Author contributions

JS: Investigation, Data curation, Visualization, Writing - original draft. PC: Formal analysis, review & editing, Visualization. PM: Formal analysis, Visualization. RM: Data curation, Writing - original draft. KT: Resources. MJ: Resources. RP: Conceptualization, Methodology, Supervision, Writing - review & editing, Funding acquisition.

## Footnotes

^1^ https://sapac.support.illumina.com/sequencing/sequencing_software/bcl2fastq-conversion-software.html

^2^ http://abseq-ref-gen.genomics.bd.com/

^3^ https://satijalab.org/seurat/articles/multimodal_vignette.html

^4^ https://zenodo.org/records/10443214
