# Supplementary material for: Dynamics of Whole Transcriptome Analysis (WTA) and Surface markers expression (AbSeq) in Immune Cells of COVID-19 Patients and Recovered captured through Single Cell Genomics
Source: Front Med (Lausanne). 2024 Jan 31;11:1297001. doi: 10.3389/fmed.2024.1297001 (PMC10864604; doi:10.3389/fmed.2024.1297001)
Supplement: Supplementary file 2 [file Table_2.pdf]

Supplementary Table S2 : List of surface marker-specific antibodies and attached oligo

Sl No	Surface Markers	Oligo sequence	Material number
1	CD195:2D7|CCR5|AHS0070	ATGGTTTAGTCGTACGTGGGT TTAGATTG GCGGTGC	940050
2	HLA-DR|CD74|AHS0035	TGTTGGTTATTCGTTAGTGCAT CCGTTTG GGCGTGG	940010
3	CD40|CD40|AHS0117	GGTGTAATTGGGCTAGAACGT ATATGCG GTAAGGCG	940049
4	CD184|CXCR4|AHS0060	CAGTGTTTAGAGCGGGTTGC ATATGTCG TTTAGAGG	940056
5	CD49d|ITGA4|AHS0063	TAGGGTGACTTAGCGATTGAT GCGTATG TTTGGGCG	940059
6	TCR-alpha_beta|TRA_TRB|AHS0078	TTGCGTCGGATTATTAGTTCG GGTATTAT GCGGTGC	940074
7	CD152|CTLA4|AHS0017	TAGTATCCGTAGTAGTTATCTG CCCGTTC GTTATGC	940034
8	CD95|FAS|AHS0023	GGCCCGTTAGAGTTGGTATCC GTATGAA GGTTAGCT	940037
9	CD127|IL7R|AHS0028	AGTTATTAGGCTCGTAGGTAT GTTTAGGT TATCGCG	940012
10	CD25:2A3|IL2RA|AHS0026	AGTTGTATGGGTTAGCCGAGA GTAGTGC GTATGATT	940009
11	CD62L:DREG-56|SELL|AHS0049	ATGGTAAATATGGGCGAATGC GGGTTGT GCTAAAGT	940041
12	CD3:UCHT1|CD3E|AHS0231	AGCTAGGTGTTATCGGCAAGT TGTACGG TGAAGTCG	940307
13	GITR|TNFRSF18|AHS0104	TCTGTGTGTCGGGTTGAATCG TAGTGAGT TAGCGTG	940096
14	CD19:SJ25C1|CD19|AHS0030	TAGTAATGTGTTCGTAGCCGG TAATAAT CTTCGTGG	940004
15	CD194|CCR4|AHS0038	AATATTAGTGGGTCCTCGCGT TGGCCGG TTGTTAGT	940047
16	CD161:DX12|KLRB1|AHS0002	GTTATGGTTGTCGGTAGAGTA TCGTGTTG CGTTAGT	940070

**17**	LAG-3|LAG3|AHS0018	CGGCATGAATTAGGCGAGACT TAGTATA CGAGCTGG	940080
**18**	CD183|CXCR3|AHS0031	AAAGTGTTGGCGTTATGTGTT CGTTAGCG GTGTGGG	940030
**19**	CD39|ENTPD1|AHS0006	TGTTGTCTTGTACGGCTTGAG TCGGGTTA ATTCGGG	940073
**20**	CD45RA:HI100|PTPRC|AHS0 009	AAGCGATTGCGAAGGGTTAG TCAGTACG<br>TTATGTTG	940011
**21**	CD7|CD7|AHS0043	GTATGTAGGTCTTATGTGTTG GCGTAGTA TGCGTTT	940029
**22**	CD16:3G8|FCGR3A|AHS0053	TAAATCTAATCGCGGTAACAT AACGGTG GGTAAGGT	940006
**23**	CD27:M-T271|CD27|AHS0025	TGTCCGGTTTAGCGAATTGGG TTGAGTC ACGTAGGT	940018
**24**	CD197|CCR7|AHS0007	AAGGGTTGTAAGTTAGTCGAT CCGCGTA TTGTCATG	940014
**25**	CD154|CD40LG|AHS0077	TAAGAGGTAAGTGCATTCGG GTATAGGC GTGATTTG	940053
**26**	CD294|PTGDR2|AHS0106	TTAGAGTTCGTGAGAGGGTA GATCGCGT TTGTAGCC	940098
**27**	Tim3|HAVCR2|AHS0016	TAGGTAGTAGTCCCGTATATC CGATCCGT GTTGTTT	940066
**28**	CD5:UCHT2|CD5|AHS0047	ACGAAGCGAGCGAAGAACCT ATGCGATT GAGTAAGT	940038
**29**	CD14:MPHIP9|CD14|AHS003 7	TGGCCCGTGGTAGCGCAATGT GAGATCG<br>TAATAAGT	940005
**30**	CD8:RPA-T8|CD8A|AHS0027	TGATTGGGTACGCGCTTGGCT TATATAGT CGGGTCT	940003
**31**	CD56:NCAM16.2|NCAM1|AH S0019	AGAGGTTGAGTCGTAATAATA ATCGGAA GGCGTTGG	940007
**32**	CD21:B-LY4|CR2|AHS0074	GTATTCGCGTATTGTCAGTCG GTAGGGTT ATGGTCT	940048
**33**	CD279:EH12-1|PDCD1|AHS0014	ATGGTAGTATCACGACGTAGT AGGGTAA TTGGCAGT	940015
**34**	CD45|PTPRC|AHS0040	GTGCGAAATGGCGGAATGTTA TCTGCGA ATGTAGTC	940002

**35**	CD69|CD69|AHS0010	CAATAACGGGTCATAGTAAGT CGCGAGT AAGAGGGC	940019
**36**	CD28:CD28.2|CD28|AHS0024	TTGGTTTCGTAAGCGGCTAAG CGTATCTC GTGTTTG	940017
**37**	CD38:HIT2|CD38|AHS0022	GTCAACGATGGGTAGCGGTA GAAATAAC GGAACTGG	940013
**38**	TCR-gamma delta:B1|TRD_TRG|AHS0015	GATTCTTATAGTCGTTGCGTA GGTTCGTC TGTGAGT	940057
**39**	CD4:SK3|CD4|AHS0032	TCGGTGTTATGAGTAGGTCGT CGTGCGG TTTGATGT	940001
**40**	CXCR5|CXCR5|AHS0039	AGGAAGGTCGATTGTATAACG CGGCATT GTAACGGC	940042
