# Supplementary material for: Dynamics of Whole Transcriptome Analysis (WTA) and Surface markers expression (AbSeq) in Immune Cells of COVID-19 Patients and Recovered captured through Single Cell Genomics
Source: Front Med (Lausanne). 2024 Jan 31;11:1297001. doi: 10.3389/fmed.2024.1297001 (PMC10864604; doi:10.3389/fmed.2024.1297001)
Supplement: Supplementary file 3 [file Table_3.pdf]

Supplementary Table S3: Cell types and corresponding AbSeq/WTA markers used for identification.

S.no	Cell type	AbSeq (Surface marker sequencing)	WTA (Whole Transcriptome Analysis)
1.	Activated CD4+ T cell	CD19-,<br>CD38,<br>CD4+,<br>CD45RA,<br>CXCR3	CD3E<br>IL7R
2.	CD4+ TCM	CD27,<br>CD4+,<br>CD45RA,<br>CD62L	CD8A<br>IL7R
3.	CD8+TCM	CD8A	CD8A
4.	CD8+TEM	CD16<br>CD3E	IL7R
5.	Classical Switch Memory B cells (CSMB)	CD19,<br>CD27	
6.	Classical Monocytes	CD14,<br>CD16	HLA-B<br>SRGN
7.	Dendritic Cells	CD45RA<br>CD8A	LY86<br>BDCA1<br>SRGN
8.	Intermediate Monocytes	CD16+	HLA-B
9.	Naive B	CD19+,<br>CD27<br>CD38	MS4A1<br>HMGB2<br>IGHA1
10.	NKT		
11.	NK	CD8A,<br>ZNF683<br>CD19,<br>CD3E,<br>CD56	NKG7<br>GNLY<br>SRGN
12.	Naive CD4+/CD8+ T	CCR7,<br>CD27	CD8A

13.	Proliferating NK	CD56, CD16	CD127
14.	Plasmablast		IGHG1
15.	Platelets		PPBP
16.	Th1	CD4+ CD45RO, CXCR3	CD4+
17	Th2	CD3E,<br>CD4+,<br>CCR4	
