# Supplementary material for: Dynamics of Whole Transcriptome Analysis (WTA) and Surface markers expression (AbSeq) in Immune Cells of COVID-19 Patients and Recovered captured through Single Cell Genomics
Source: Front Med (Lausanne). 2024 Jan 31;11:1297001. doi: 10.3389/fmed.2024.1297001 (PMC10864604; doi:10.3389/fmed.2024.1297001)
Supplement: Supplementary file 8 [file Table_8.pdf]

Supplementary Table S8 - Table contains surface markers and their aliases names, cell type information, general function and role in infectious diseases.

S.No	Marker Name	Aliases Names	Associated Immune Cells	General Function	Role in infection	Ref.
1	CD19	B4, Blymphocyte antigen CD19	B cells	B cell marker involved in B cell developm ent and activation.	The expression of CD19 was notably decreased in nearly all subsets of B cells among individuals who were infected and recovered from the infection.	PMID: 34552055
2	CD14	LPS receptor, monocyte differentiation antigen CD14	Monocytes and macrophages	Monocyte markers are involved in the innate immune response.	A larger N-terminal pocket and several rim residues on CD14 are thought to be crucial for ligand binding and cell activation.	PMID: 23898465
3	CD28	Tp44, Tcellspecific surface glycoprotein CD28	T cells	The costimulatory molecule on T cells for T cell activation and survival	Proliferation, survival, and CD4+ T cell activation depend on CD28 signalling (Harding et al., 1992).	PMID: 1313950
4	FAS	APO-1, TNFRSF6, CD95, apoptosis antigen 1	Activated T and B cells	Mediates apoptosis (cell death) in activated immune cells.	When T-cells are activated, CD95 is known to be increased and can indicate a tendency for apoptosis.	PMID: 32679621

**5**	FCGR 3A	CD16, Fc gamma receptor IIIA	Natural Killer (NK) cells	Fc gamma receptor on immune cells involved in antibodydependent cellular cytotoxicit y.	CD16 plays a crucial role in enabling natural killer (NK) cells to participate in antibody-dependent cellular cytotoxicity (ADCC)	PMID: 27670 158

**6**	CD38	T10, ADP-ribosyl cyclase 1, cyclic ADP-ribose hydrolase 1	Plasma cells and activated T and B cells	Enzyme involved in NAD metabolis m and cell activation.	CD38 is involved in cell control, differentiation, recruitment of effector cells during inflammation, cytokine release, and NAD availability.	PMID: 33329 591
**7**	ENTP D1	CD39, ectonucleo side triphosphat e diphospho hydrolase 1	Regulato ry T cells and activated T cells	Ectonucle oside triphospha te diphospho hydrolase 1, is involved in immune cell activation and regulation.	ENTPD1/CD39, an ectonucleotidase mainly involved in the scavenging of pro-inflammatory ATP and ADP into AMP	PMID: 34608 452
**8**	ITGA 4	CD49D, integrin alpha-4	T cells and monocyt es	Involved in cell adhesion and migration	Promote the development of regulatory B cells in peripheral immunological organs and their management of the pathogenic T cell response	PMID: 27016 608

**9**	SELL	CD62L, L-selectin, LECAM1, lymph node homing receptor	T cells, B cells, and thymocytes	Adhesion molecule involved in lymphocyte homing to lymph nodes.	The ability of Agactivated T cells to remove pathogen from peripheral locations is determined by the presence of CD62L on these cells.	PMID: 18097020
**10**	CD69	CLEC2C, early activation antigen CD69	T and B cells, NK cells, and neutrophils	Early activation marker on lymphocytes and NK cells.	The differentiation of regulatory T (Treg) cells is controlled by CD69.	PMID: 28475283
**11**	CXCR4	CD184, CXC chemokine receptor 4	T cells, B cells, monocytes, dendritic cells	Chemokin e receptor involved in cell migration and homing	CXCR4 plays a role in cellular processes, immunological responses, and growth and development	PMID: 34386499

**12**	CCR4	CD194, chemokine receptor 4	T cells, regulatory T cells, Th2 cells	Chemotaxis and migration of immune cells, regulation of Th2 response.	CCR4 and its ligands CCL17 and CCL22, which are postulated to play key roles in the pathogenesis of allergic asthma	PMID: 19630858
**13**	CCR5	CD195, chemokine receptor 5	T cells, monocytes, macrophages	Chemotaxis and migration of immune cells, involved in inflammatory responses.	CCR5 responsible for mediating chemotactic activity in leukocytes, which in turn mediates immunological and inflammatory responses,	PMID: 29163468

**14**	CCR7	CD197, chemokine receptor 7	T cells, B cells, dendritic cells	Mediates homing of immune cells to lymphoid tissues, regulates T cell activation and migration.	Performs significant functions in immune monitoring and immune cell trafficking in different tissue compartments during inflammation.	PMID: 22533 989
**15**	IL7R	CD127, interleukin -7 receptor	T cells, B cells, NK cells, dendritic cells	Receptor for interleukin -7, crucial for T cell developm ent and homeostas is.	The survival of both memory and naive T cells is promoted by IL7R	PMID: 19380 817
**16**	IL2RA	CD25, interleukin -2 receptor alpha	Regulato ry T cells, activated T cells	Receptor for interleukin -2, important for T cell activation and proliferati on.	Aside from its essential role in immune responses, the IL-2 - IL-2R pathway also contributes to the maintenance of selftolerance.	PMID: 32619 646

**17**	CXCR 3	CD183, chemokine receptor 3	T cells, NK cells	Chemotax is and migration of immune cells, are involved in Th1 response and inflammat ion.	Following activation, after infection CXCR3 is quickly upregulated on naive cells and preferentially remains strongly expressed on Th1-type CD4+ T cells.	PMID: 21376 175

**18**	LAG3	CD223, lymphocyte activation gene 3	T cells, regulatory T cells, B cells	Negative regulator of T cell activation modulates immune response and tolerance.	LAG-3 plays a crucial regulatory role in the immune system, affecting immunological responses and preserving immune homeostasis.	PMID: 34067904
**19**	PDCD1	PD-1, CD279, programmed cell death protein 1	T cells, B cells	Inhibitory receptor on T cells regulates immune response and prevents autoimmunity.	PD-1 acts as an immune checkpoint molecule, dampening T cell activity to prevent potential autoimmune tissue damage during immune responses.	PMID: 32265932
**20**	PTGDR2	CRTH2, CD294, prostaglandin D2 receptor 2	Th2 cells, eosinophils, basophils	Mediates allergic responses, involved in eosinophil and basophil activation.	It synthesizes prostanoids that facilitate the body's responses to various physiological stresses, including infection and inflammation.	PMID: 28261111
**21**	PTPRC	CD45, protein tyrosine phosphatase, receptor type C	T cells, B cells, NK cells, monocytes, dendritic cells	Protein tyrosine phosphatase receptor, involved in signalling and activation	PTPRC is found only in cells belonging to different hematopoietic lineages and plays a critical role in controlling important processes, such as B- and T-cell receptor signalling.	PMID: 28941747
				of immune cells		

**22**	TRDC	TCRD, T cell receptor delta chain	T cells, specifica lly gamma-delta T cells	Associate d with gamma-delta T cells, plays a role in innate and adaptive immunity.	γδ T cells have the ability to recognize antigens.	PMID: 30116 753
**23**	CD27	TNFRSF7, Tumor necrosis factor receptor superfamil y member 7	B cells, T cells, and natural killer (NK) cells.	Plays a role in T and B cell activation, differentia tion, and memory formation.	The CD27/CD70 pathway is important for the formation of germinal centres, B cell activation, and the production of neutralising antibodies.	PMID: 20699 361
**24**	CD4	T4, Leu3, T-cell surface glycoprotei n CD4	helper T cells (CD4+ T cells), macroph ages and dendritic cells.	Essential for the activation and regulation of helper T cells	CD4+ T cells serve as essential helpers, the production of antibodies by B cells and playing a crucial role in the development of cytotoxic and memory CD8+ T cell populations.	PMID: 22266 691
**25**	CD40	TNFRSF5, Tumor necrosis factor receptor superfamil y member 5	B cells, dendritic cells.	Facilitates interaction between B cells and T cells, promoting immune responses.	The role of costimulatory molecules in both immune responses and autoimmune reactions is widely known.	PMID: 29988 701,P MID: 36420 488

**26**	CD40 LG	CD154, TNFSF5, CD40 ligand	Activated CD4+ T cells.	Engages CD40 on B cells, providing vital signals for B cell activation and antibody production	CD40L plays a crucial role in regulating both cellular and humoral immune responses during viral immunopathogenesis.	PMID: 34898656

**27**	CD5	T1, Leu1, T-cell surface glycoprotei n CD5	T cells, B cells, and natural killer T (NKT) cells.	Modulates T and B cell activation and regulates immune responses	CD5 also acts as a scavenger-like receptor and participates in regulating cell death. It can also serve as a receptor for pathogenassociated molecular patterns.	PMID: 21482089
**28**	CD7	TP41, T- cell surface antigen Leu-9, Tcell antigen CD7	T cells, NK cells, B cells	Involved in T cell developm ent and activation	CD7, a molecule expressed early in the development of T lymphocytes, and is essential for boosting the activation of T cells.	PMID: 7506726
**29**	CD8A	CD8, T- cell surface glycoprotei n CD8 alpha chain	Cytotoxic T cells (CD8+ T cells),	Recognizes and interacts with antigens presented by major histocompatibility complex (MHC) class I molecules.	CD8+ T cells and antibodies in combination may provide the most effective form of protective immunity.	PMID: 15140950

**30**	GITR	TNFRSF18, Glucocorticoidinduced TNFRrelated protein	T cells (Tregs), activated CD4+ and CD8+ T cells	Regulates immune responses and T cell activation, playing a role in immune tolerance and suppression.	GITR has an inherent function in supporting the survival of CD8 T cells with T-cell receptor (TCR).	PMID: 25590581
**31**	HAVCR2	TIM-3, Tcell immunogl obulin and mucin domaincontaining protein 3	Activated T cells, natural killer (NK) cells, DCs, and macrophages.	Regulates immune responses, particularly in T cells and macrophages, and can modulate	HAVCR2 increases immunological tolerance, controls macrophage activation	PMID: 15140950

**32**	CD3E	T3E, CD3 epsilon chain	T cells	Plays a crucial role in T cell receptor signaling	CD3E plays a critical role in connecting antigen recognition to intracellular signal transduction pathways.	PMID: 32730808
**33**	CR2	CD21, Complement receptor type 2	B cells	B cell activation and regulation	CR2 plays significant roles in the emergence of autoimmunity as well as typical humoral immune responses.	PMID: 10809953

				T cell exhaustion.		

**34**	CXCR 5	CD185, CX-C chemokine receptor type 5	B cells and follicular helper T cells,	B cell migration and germinal centre formation.	Facilitates B and follicular helper T (TFH) cell homing into secondary lymphoid organ follicles.	PMID: 31028 278
**35**	NCA M1	CD56, Neural cell adhesion molecule 1	Natural killer (NK)	Cell adhesion and signaling.	CD56 appears to be restricted to immune cells that have been activated and demonstrate some degree of cytotoxicity.	PMID: 28791 027
**36**	TRAC	TCR- alpha, Tcell receptor alpha chain	T cells	Encodes the T cell receptor alpha chain.	Trac aids in the generation of memorylike adaptive responses.	PMID: 33183 352
**37**	CTLA 4	CD152, Cytotoxic Tlymphocyt eassociated protein 4	Activate d T cells and regulator y T cells	Regulatin g immune responses by inhibiting T cell activation.	CTLA-4 is known as inhibitory immune checkpoint molecule	PMID: 33508 291
**38**	KLRB 1	CD161, Killer cell lectinlike receptor B1	Natural killer (NK) cells	Involved in immune cell activation and cytotoxicit y.	KLRB1 homing molecules and contribute to the migration and retention of T cells subsets.	PMID: 24987 392
**39**	CD74	HLA-DR antigensassociated invariant chain	APCs ,B cells and macroph ages	Participati ng in antigen presentati on and	CD74 plays a role in infection by participating in antigen presentation and immune response modulation.	PMID: 36323 260
				immune responses		
