# Supplementary material for: Dynamics of Whole Transcriptome Analysis (WTA) and Surface markers expression (AbSeq) in Immune Cells of COVID-19 Patients and Recovered captured through Single Cell Genomics
Source: Front Med (Lausanne). 2024 Jan 31;11:1297001. doi: 10.3389/fmed.2024.1297001 (PMC10864604; doi:10.3389/fmed.2024.1297001)
Supplement: Supplementary file 10 [file Data_Sheet_1.pdf]

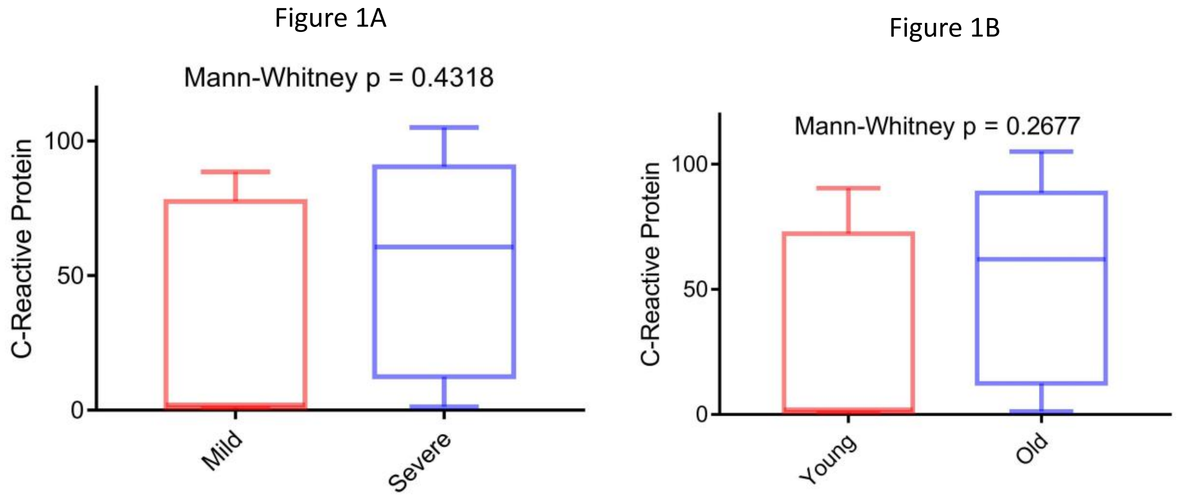

Supplementary Figure 1: C-Reactive Protein expression between Disease severity (1A) and Age group (1B) within Infected group

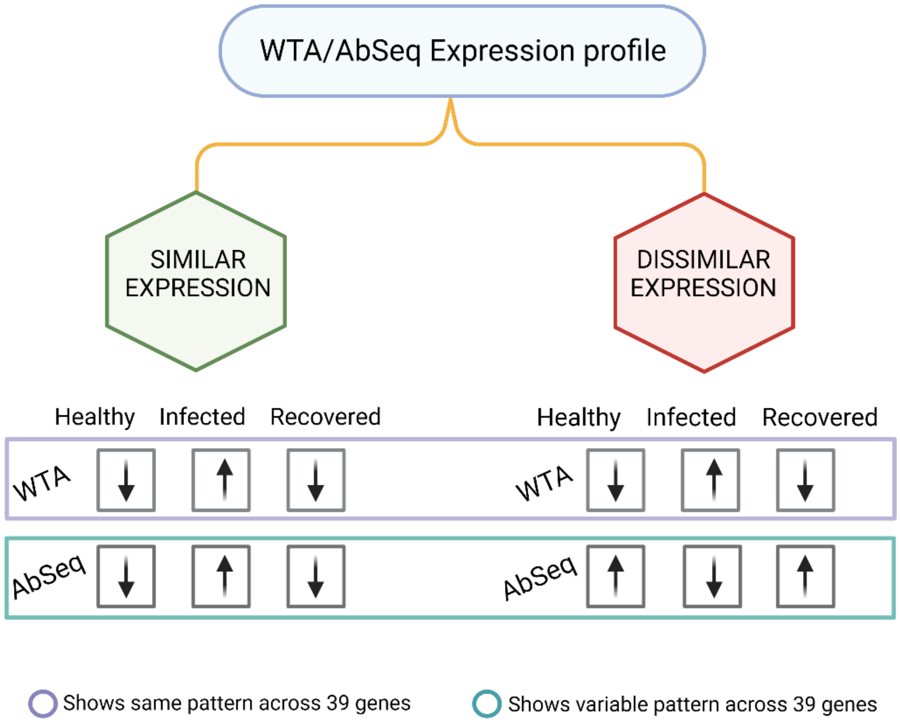

Supplementary Figure 2: Categorization of WTA/AbSeq on the basis of similarity and dissimilarity in their expression trajectories.

Patterns of WTA/AbSeq expression

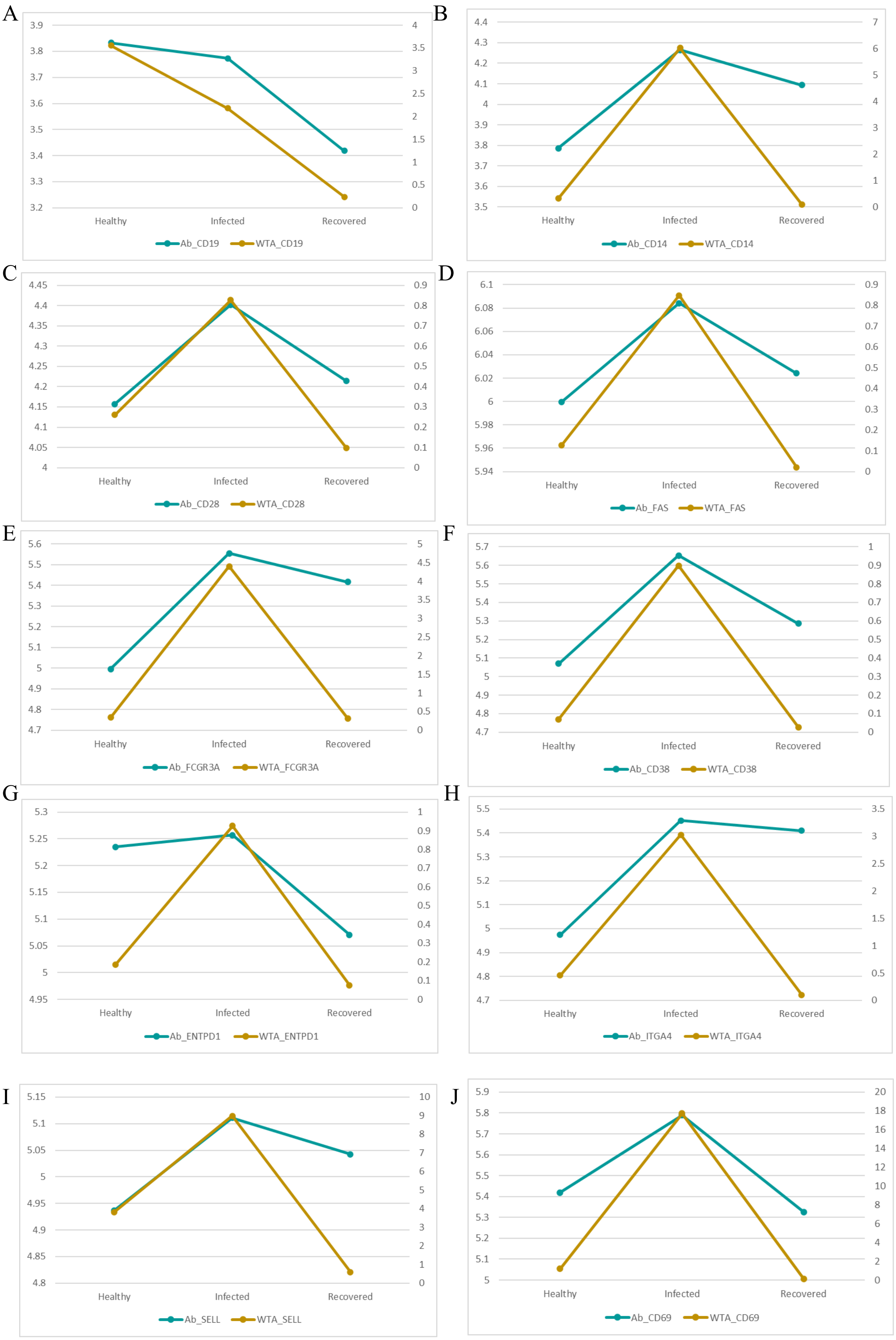

K

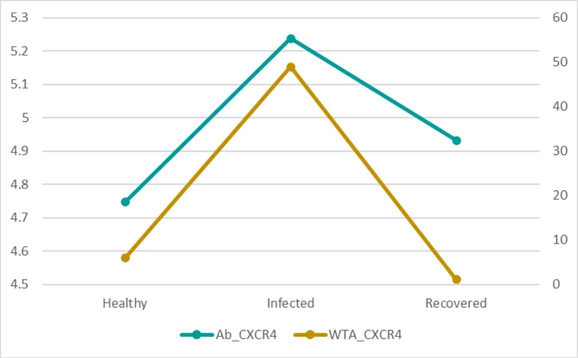

Supplementary Figure 3 (A-K): Similar pattern - parallel trajectories between WTA and AbSeq among all groups.

A

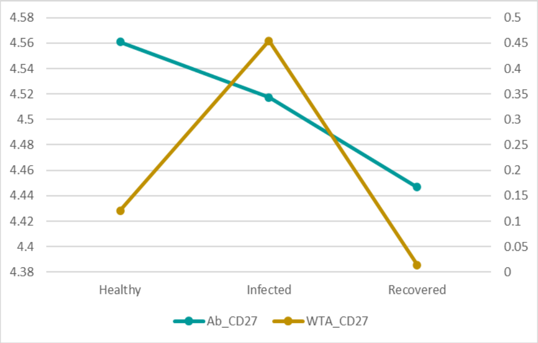

B

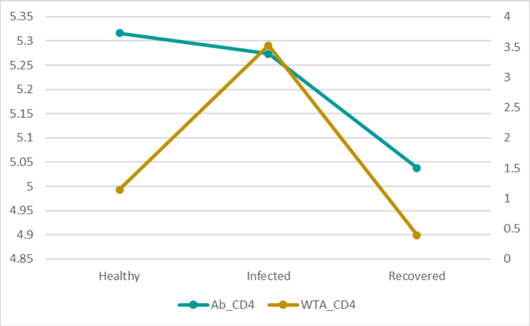

C

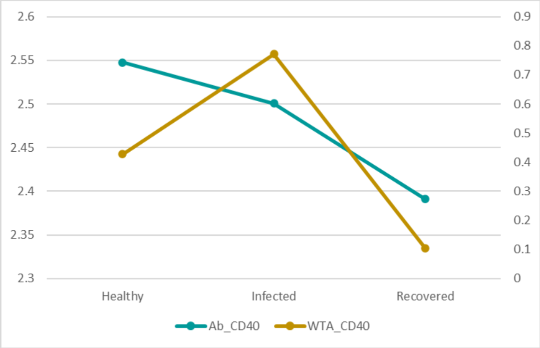

D

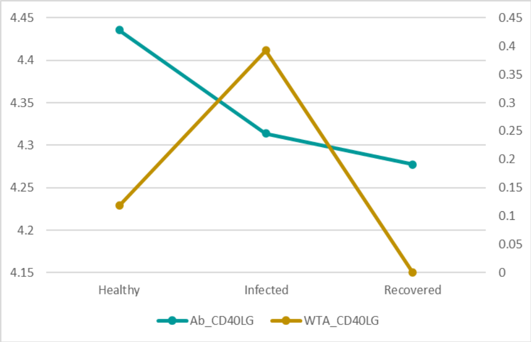

E

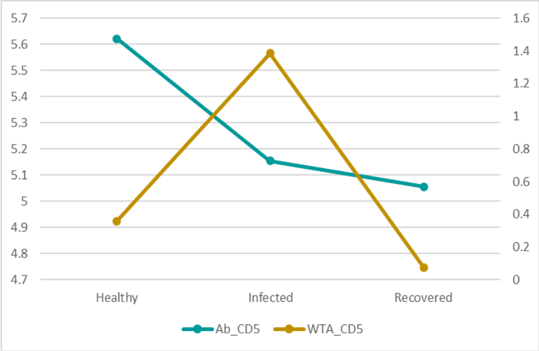

F

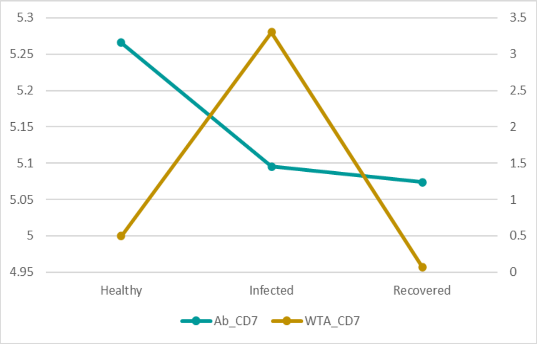

G

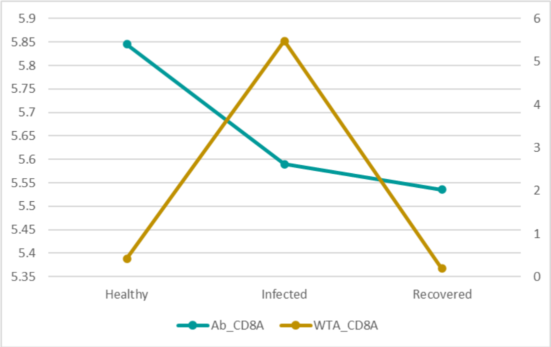

H

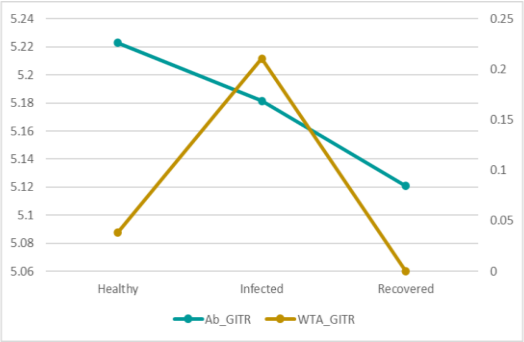

I

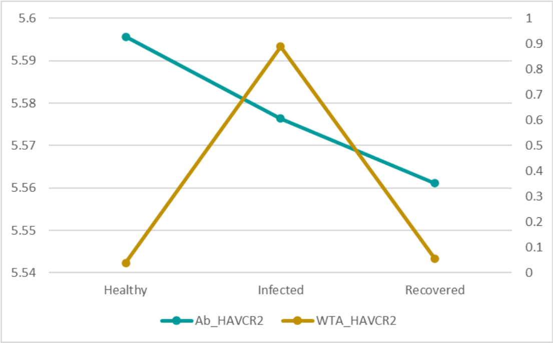

Supplementary Figure 4 (A-I): Dissimilar pattern- Opposite trajectories between WTA and AbSeq among Healthy group.

A

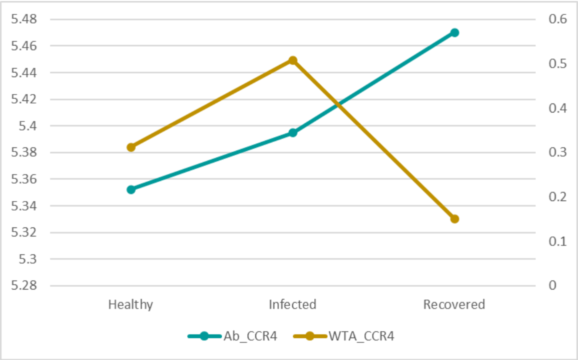

B

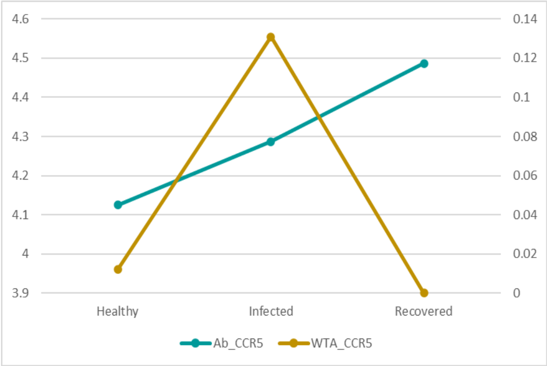

C

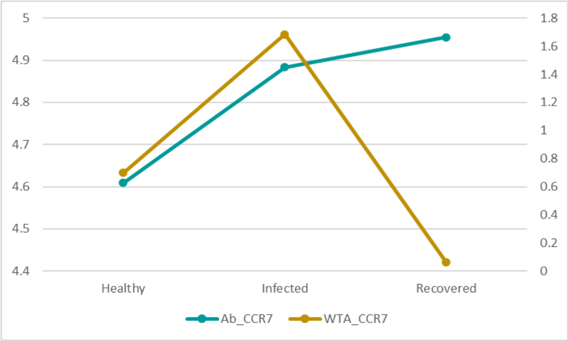

D

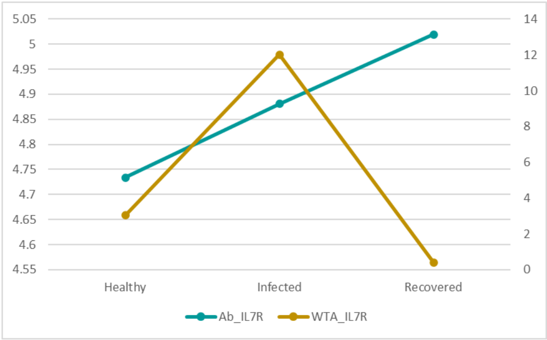

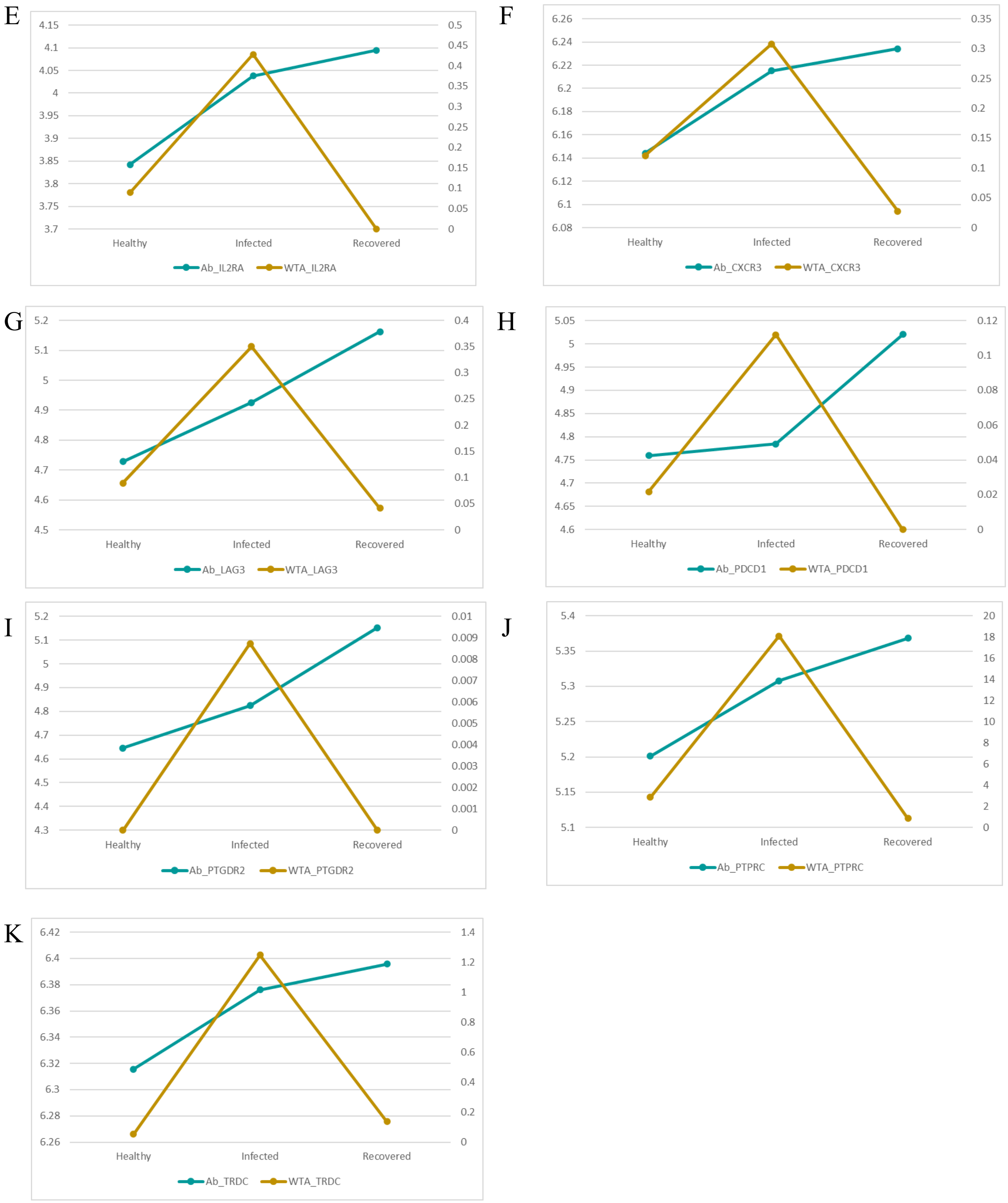

Supplementary Figure 5 (A-K): Dissimilar pattern- opposite trajectories between WTA and AbSeq among Recovered group.

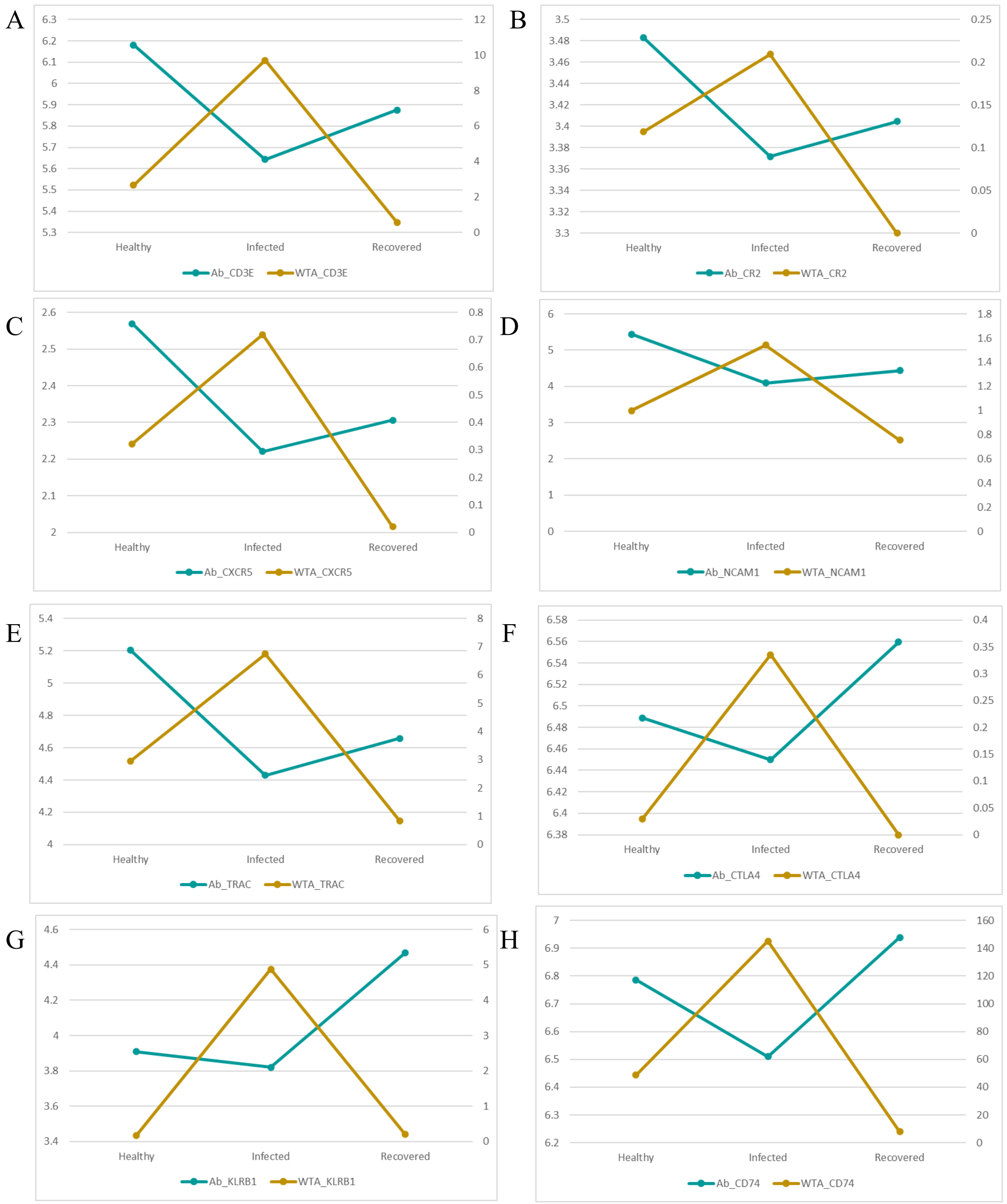

Supplementary Figure 6 (A-H): Dissimilar pattern- opposite trajectories between WTA and AbSeq among all groups.

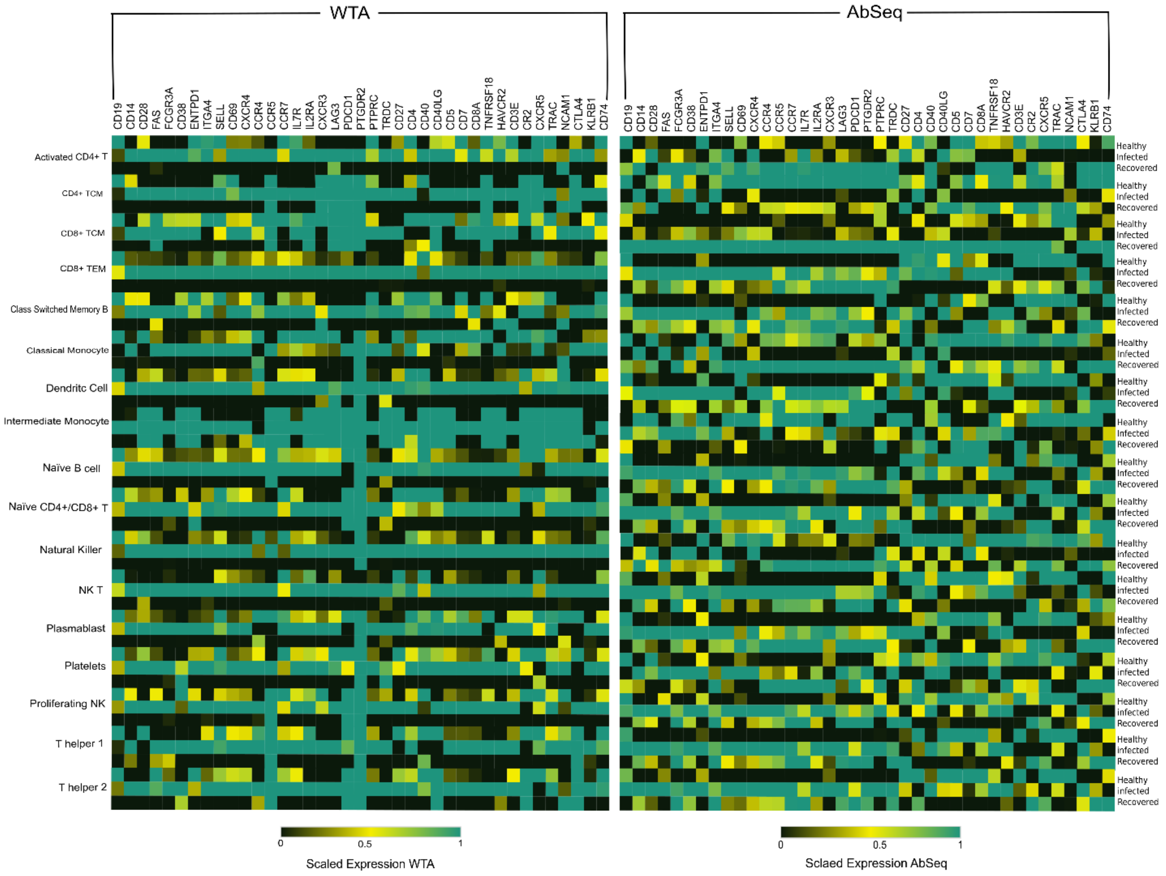

Supplementary Figure 7: WTA/AbSeq cell type-wise expression profile among healthy, infected and recovered.
